# Wild cereal harvesting ca. 14,000 years ago at Jeftelik (Western Syria)

**DOI:** 10.1371/journal.pone.0350364

**Published:** 2026-11-10

**Authors:** Khaled Abdo, Antonio Palomo Pérez, Patricia C. Anderson, Niccolò Mazzucco, Maya Haïdar-Boustani, Luis C. Teira, Juan Jose Ibáñez-Estévez

**Affiliations:** 1 Departamento de Arqueología y Antropología, Institució Milà i Fontanals, Consejo Superior de Investigaciones Científicas (IMF-CSIC), Barcelona, Spain; 2 Directorate-General of Antiquities & Museums, DGAM, Damascus, Syria; 3 Department of Prehistory, Autonomous University of Barcelona, Barcelona, Spain; 4 Univ. Nice Sophia Antipolis, CNRS, CEPAM, UMR, Nice, France; 5 Department of Civilizations and Forms of Knowledge, University of Pisa, Pisa, Italy; 6 Saint Joseph University of Beirut, Beirut, Lebanon; 7 Instituto Internacional de Investigaciones Prehistóricas de Cantabria (IIIPC), Santander, Spain; University of Haifa, Zinman Institute of Archaeology, ISRAEL

## Abstract

The harvesting of wild cereals with sickle blades during the Late Epipalaeolithic Natufian period (ca. 14.6–11.5 ka cal BP) is widely documented; however, the scale and methods of sickle harvesting remain poorly understood. This study examines plant-harvesting activities through the functional analysis of glossed lithic tools from the Early Natufian site of Jeftelik (Western Syria), dated to ca. 14,000 years ago. Twelve sickle blades recovered from secure Natufian contexts were analysed using confocal microscopy and three-dimensional surface texture analysis. Their surface textures were compared with an experimental reference collection, including tools used to harvest wild cereals in natural stands on the one hand and tools used to cut other siliceous plants, such as reeds and grasses, on the other. The results indicate that most glossed tools were used for harvesting wild cereals, although the processing of other siliceous plants was also identified. Tool-level analysis shows that five implements were primarily used for harvesting wild cereals, one mainly for cutting reeds or grasses, and six display mixed plant-processing signals. Estimates of use intensity suggest generally moderate-to-long harvesting episodes of wild cereals: two tools exhibit intermediate use, between 10 and 20 hours, whereas three tools show evidence of more prolonged use, for more than 20 hours. Glossed sickle blades represent only a small proportion of the lithic assemblage at Jeftelik (ca. 1%). These results indicate that, although wild cereals were harvested at Jeftelik sometimes with relatively high use intensity—sickle harvesting was not a frequently performed activity. Two interpretations can be proposed: either wild cereals were not intensively exploited, or, more likely, they were an important resource, but methods other than sickle harvesting were also employed. Wild cereal harvesting likely formed part of a broader and flexible exploitation system, in which cereals were collected using multiple techniques, including reaping with sickles during periods when intensified resource acquisition was required.

## Introduction

The Late Epipalaeolithic Natufian period (c. 14.6–11.5 cal BP) represents the final phase preceding the large-scale production of plant foods in the Levant. It is generally divided into two main phases: the Early Natufian, which developed under relatively warm and humid climatic conditions, and the Late Natufian, which partially overlapped with the cooler and drier environmental conditions of the Younger Dryas [1–3]. Natufian sites are widely distributed across both the northern and southern Levant and occur in a wide range of ecological settings, including coastal areas (Nahal Oren and El-Wad), riverbanks, and lake margins (Jeftelik, Mureybet, and Abu Hureyra) [4–6], as well as semi-arid environments (Bal’as, El Nadaouyieh 3, El Kowm, Kaus Kozah and Baaz) [7,8].

During this period, archaeobotanical evidence increased substantially, demonstrating the intensive use of a wide range of plant species, including the wild progenitors of the first domesticated cereals. There is broad agreement that Natufian communities engaged in the harvesting of wild cereals [4]. Most scholars argue that these cereals were harvested from natural stands, placing Natufian groups at what has been described as the “threshold to agriculture” [9]. Cereal exploitation has been closely linked to the emergence of sedentary lifeways [6,10–15]. As emphasized by Flannery (1969) [16], sedentism was likely a prerequisite for the adoption of new methods of plant processing, including harvesting, preparation, and storage.

Key technological innovations of the Natufian culture, most notably ground stone tools and sickle blades, were central to the effective exploitation of wild cereals [17,18]. Sickle blades, identifiable by the presence of a characteristic glossy sheen along their cutting edges, were used for harvesting wild plants during the Natufian period, although they are less frequent than in later Neolithic assemblages. The analysis of use-wear polish on sickle blades has long been regarded as a complementary line of evidence to archaeobotanical data in studies of the emergence of cereal exploitation and the development of agricultural economies. Early interpretations of sickle blade gloss played a particularly influential role in debates surrounding Natufian subsistence strategies. The discovery of glossed sickle blades led Dorothy Garrod [19,20] to propose that the Natufians were the earliest agriculturalists in Southwest Asia. Nevertheless, this interpretation was later abandoned following analyses of carbonized plant remains, which demonstrated that the cereals exploited by Natufian groups were morphologically wild rather than domesticated [21,22].

Importantly, however, gloss on the cutting edges of stone tools is not exclusive to cereal harvesting. The cutting of other siliceous plants—such as reeds or feather grasses (i.e., *Stipa* spp.)—can also produce glossy wear traces. These non-cereal plants were likely exploited for a variety of purposes, including the production of crafts such as basketry, nets, mats, and textiles, as well as for construction materials used in the roofing or flooring of Natufian dwellings.

This paper aims to highlight the importance of cereal exploitation alongside the use of other plant resources through the analysis of use-wear on glossed tools from the site of Jeftelik, an Early Natufian site located in the Homs Gap (Western Syria), outside the core Natufian zone. The site is situated in a Mediterranean environmental setting characterized by relatively high annual rainfall (approximately 500–600 mm) and ecological conditions favourable to the growth of a diverse range of plant species. Using confocal microscopy and quantitative texture analysis, this study examines glossed tools as direct indicators of plant-harvesting activities. These tools provide a particularly informative proxy for assessing the intensity and nature of cutting activities for plant exploitation in this Early Natufian context.

### Quantitative use-wear analysis

The development of research and analytical approaches to sickle blades has progressed through several key stages. Use-wear analysis, first introduced by Semenov (1964) [23], identifies tool function through the examination of wear traces such as micro-scars, edge rounding, striations, and use-polish. The cutting of silica-rich plants, particularly cereals, produces a distinctive polish along tool edges which, after several hours of use, becomes visible to the naked eye as a sheen commonly referred to as sickle gloss [22,24,25].

Traditional use-wear analysis relies primarily on microscopic observation and the visual comparison of wear traces on archaeological artifacts, enabling functional interpretations to be established [26,27]. More recently, significant methodological advances, most notably the application of confocal microscopy and three-dimensional surface texture analysis, have substantially transformed use-wear studies [28–35].

While traditional use-wear analysis relies on direct comparative examination, these newer quantitative approaches allow for a more precise characterization of worked materials. In the case of sickle blade polish, they make it possible to distinguish between different plant-harvesting activities based on microscopic surface features. As a result, they have considerably expanded the analytical potential of sickle blade studies and opened new perspectives on prehistoric plant exploitation [34,36–39].

Previous use-wear analyses of harvesting tools from pre-Neolithic contexts, prior to the development of our quantitative approach based on confocal microscopy, relied primarily on the visual comparison of experimental and archaeological tools. Evidence from the lakeshore site of Ohalo II demonstrates that wild cereal harvesting was practiced as early as the Last Glacial Maximum, approximately 23,000 years ago [40]

The harvesting of wild cereals during the Natufian period represents a key stage in the long transition from plant gathering to cultivation in Southwest Asia. Use-wear studies of glossed flint blades indicate that Natufian groups regularly cut silica-rich plants, particularly wild cereals, using composite sickles. Unger-Hamilton’s experimental work demonstrated that the characteristics of sickle gloss vary according to harvesting conditions and use intensity, and that glossed blades from Natufian and Early Neolithic contexts can be related to the harvesting of wild cereals and other grasses [41]. Patricia Anderson’s experimental cultivation and harvesting of wild einkorn further showed that cutting green or semi-ripe wild cereals produces distinctive microwear traces comparable to those observed on Natufian sickle blades. These experiments suggest that Natufian communities often harvested wild cereals before full ripening, when the grains were still attached to the rachis and could be collected efficiently before natural seed dispersal [42].

More recent quantitative texture analyses have confirmed that Natufian cereal harvesting mainly targeted wild cereals growing in natural stands [38]. These cereals were generally cut high on the stem, close to the ear rather than near the ground, indicating that the primary objective was grain collection rather than the systematic acquisition of straw [43].

### Archaeological site and lithic material

The site of Jeftelik is located west of Homs, near the northern edge of the Bouqaia Basin, close to the course of the Al Raouil River (34.786214° N, 36.318550° E; WGS84) (Fig 1). The site dates to the Early Natufian period and was discovered in 2004 by the Syrian–Lebanese–Spanish mission under the supervision of Michel al-Maqdissi (DGAM, Syria), Maya Haïdar-Boustani (Saint Joseph University of Beirut), and Juan José Ibáñez (Spanish National Research Council – CSIC) [44,45].

**Fig 1:**
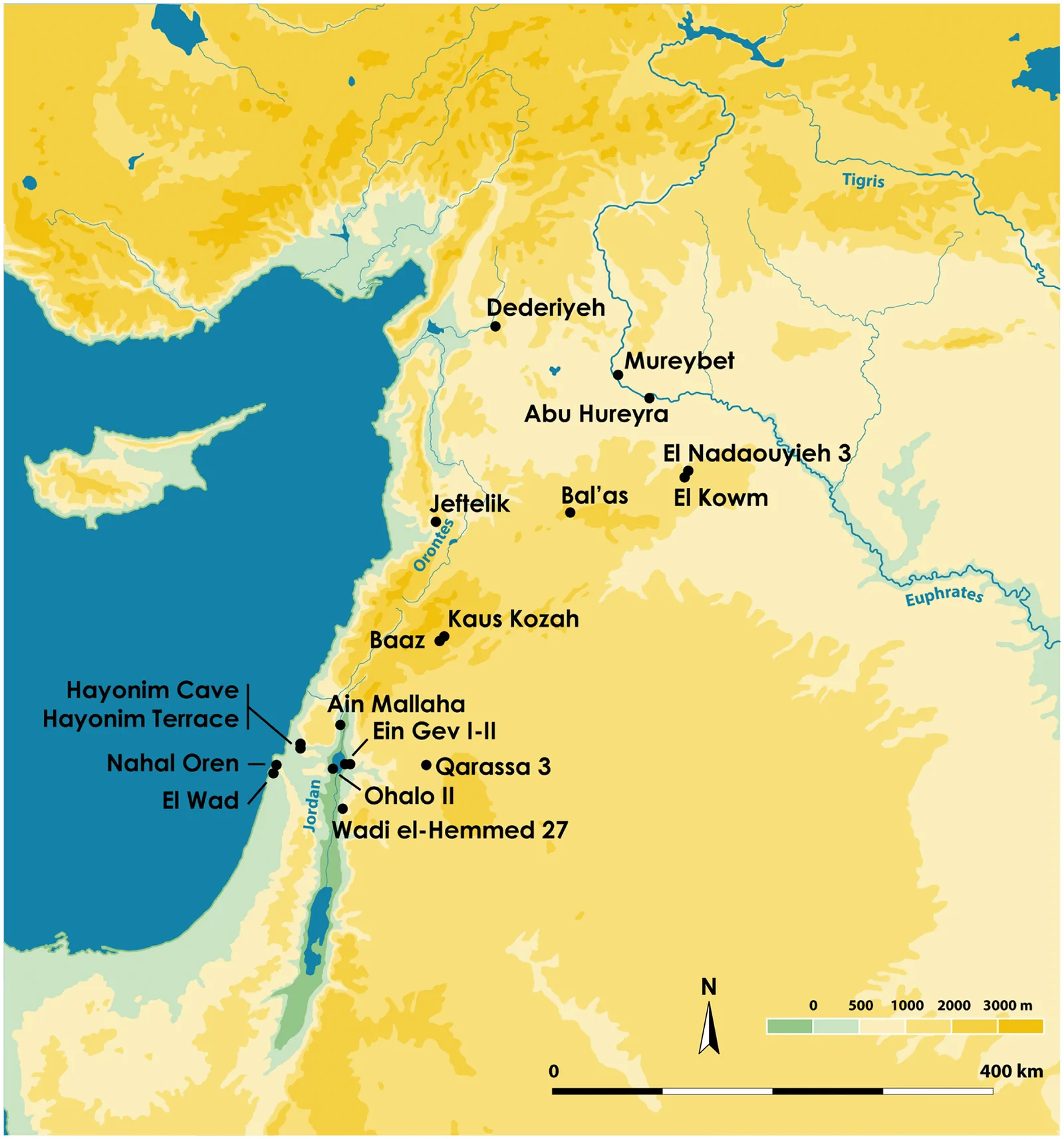
Map showing the location of the Jeftelik site in relation to the main Natufian sites in the region.

Jeftelik is situated in the Homs Gap, which serves as a crucial communication corridor between coastal regions and inland Syria [44]. Excavations conducted between 2008 and 2010 uncovered several Natufian stone structures that had been partially disturbed by Bronze Age, Byzantine, and Ottoman-period pits. Among the discoveries was a large circular structure with a diameter of approximately 6 m (around 28 m^2^). The walls of this structure slope outward, supporting a truncated conical platform about 70 cm high. In addition, the site features several circular installations, as well as semi-circular and other less clearly defined structures located to the west, some of which are in close proximity to the main structure (Fig 2). Radiocarbon analyses of samples from the site yielded dates ranging between the end of the 15th and the beginning of the 14th millennium cal BP [15,46].

**Fig 2:**
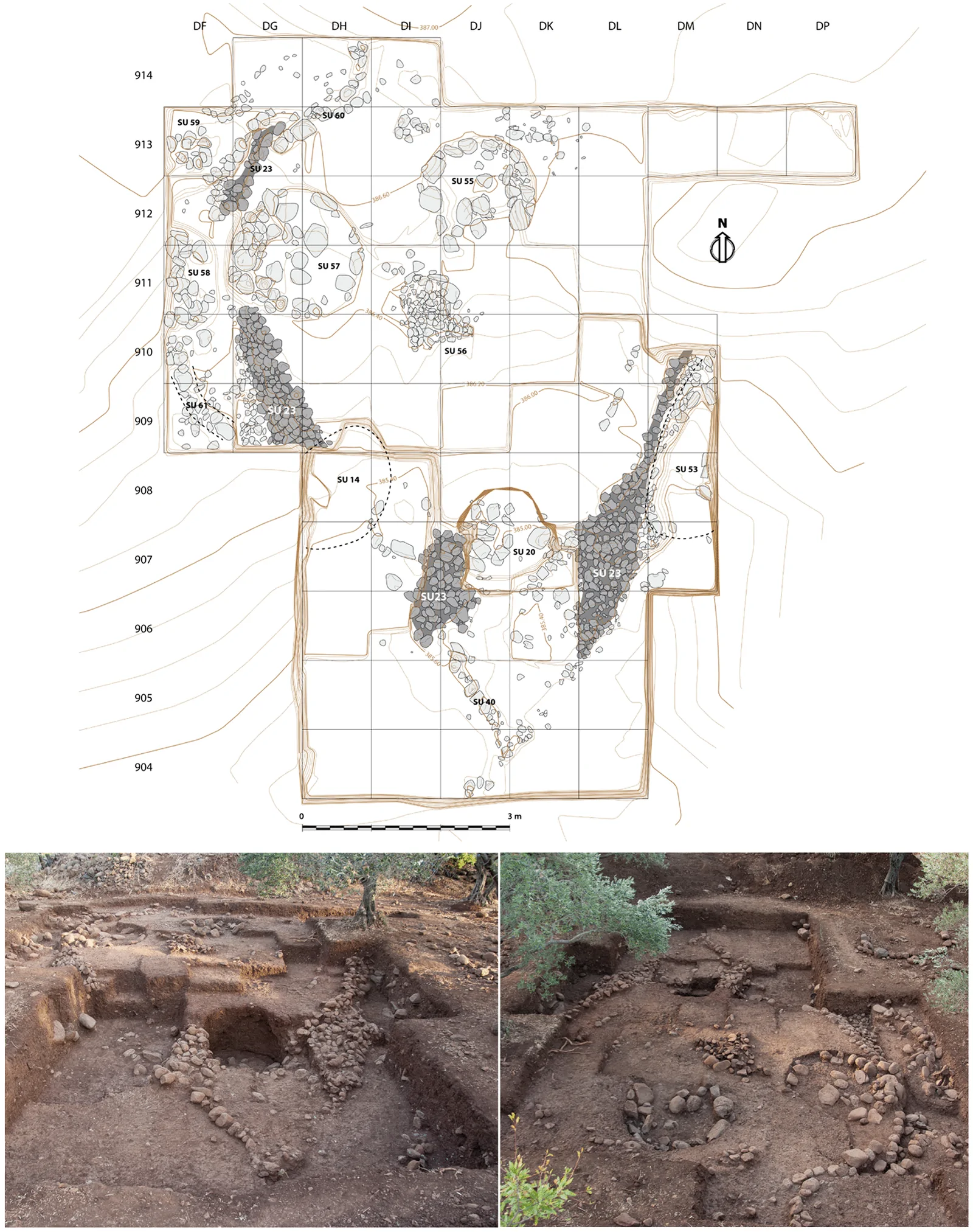
Plan and excavation views of Natufian circular structures at Jeftelik.

The site’s Natufian lithic industry is dominated by fine-grained local flint sourced from Marmarita (~6 km away), with minimal obsidian presence. Knapping focused on producing small- to medium-sized elongated blanks from unipolar and multi-platform cores. Among the retouched tools, endscrapers are dominant (17.3%), followed closely by diagnostic Natufian lunates (14.7%). In an analysed sample of 75 lunates, 62% show Helwan retouch, 34% abrupt retouch, and 4% both. Functional analysis of the lunates currently underway indicates that they were used as projectile inserts [47]. The remaining assemblage includes drills, burins, and a low proportion of sickle blades, which constitute the focus of the present study, are represented in very low proportions (1% of the retouched tool assemblage). The ground stone assemblage consists of querns, mortars, handstones, pestles, and a half recipient-mortar. In addition, several decorated objects were also discovered at Jeftelik [45,46].

In traditional typological studies of Natufian and Neolithic lithic assemblages, flakes and blades displaying macroscopic gloss are generally classified as sickle blades. Use-wear analysis has refined this interpretation by demonstrating that macroscopic gloss is not exclusive to cereal harvesting but may also result from other technical activities, such as cutting other siliceous plants, use as threshing-sledge elements, or working mineral materials in the presence of water. Experimental studies have shown that macroscopic gloss develops on plant-cutting tools after relatively short periods of use (approximately one hour). Consequently, the presence of macroscopic gloss provides an efficient criterion for selecting tools used in plant cutting, although implements used only for very short periods are unlikely to develop visible gloss and therefore remain undetected. Subsequent microscopic use-wear analysis allows gloss produced by plant cutting to be distinguished from gloss resulting from other activities, such as plant scraping, use in threshing sledges, or the working of mineral materials in the presence of water.

In order to recover the sickle blades, approximately 70% of the lithic assemblage bags recovered during the three excavation seasons were reopened. These materials were stored in the storage facilities of the Al-Mashrafa site (Qatna), located in the eastern rural area of Homs, where the selection took place. All blades and flakes were systematically re-examined to identify as many sickle blades as possible, particularly those bearing clearly visible use-wear polish along one of their edges, observable with the naked eye. Because of this extended review, sixteen sickle blades were identified. However, the present study focuses on the analysis of twelve glossed tools selected exclusively from secure Natufian levels in order to avoid potential errors resulting from stratigraphic mixing.

The analyses were carried out at the IMF (Institució Milà i Fontanals) in Barcelona, part of the Spanish National Research Council (CSIC). All necessary permits were obtained for the described study, which complied with all relevant regulations, following official authorization from the Directorate-General of Antiquities and Museums (DGAM) in Damascus for the transfer of the material to Spain. The study was conducted in two stages: two sickle blades were analysed in 2024, and the remaining ten were studied in 2025.

The analysed sample reveals the use of a variety of blanks for the production of sickle blades. Nearly half of the specimens were manufactured on blades (five examples) or bladelets (six examples), while a single specimen was made on a flake. These blanks were primarily straight and derived from unipolar core reduction strategies, with the dorsal faces exhibiting between one and three parallel blade scars from prior removals, although subsequent intensive retouch may have obscured additional scar patterns. Morphologically, the majority of the sickle inserts either naturally feature parallel lateral edges or achieved parallelism through marginal retouch, with the exception of only four non-parallel examples. The tools range from 19.5 to 48.1 mm in length and 6.5 to 20.9 mm in width. The preservation of cortex is generally absent across the assemblage; however, two specimens are classified as lateral blades, with both retaining remnants of calcareous cortex only along one margin. Retouch on the back opposite the glossed edge is predominantly abrupt and direct, observed on six sickle blades. Two examples display Helwan-type retouch, while one specimen bears covering direct simple retouch. In three cases, the dorsal edge was left unmodified. Modifications of the active edge were documented on two sickle blades, consisting of the creation of small notches along the same polished edge. Furthermore, three sickle blades exhibit modification of a broken end through truncation produced by abrupt retouch, either direct or inverse, possibly to regularize the insert for hafting purposes (Table 1). The study of polish distribution along the edges of the sickle blades from the Jeftelik site indicates a clear predominance of straight hafting positions, with only slight inclinations observed in some cases. The analysis of the fracture-polish relationship on the sickle blades showed that fracturing occurred prior to use in five cases and post-use in two cases. The remaining five instances exhibited an ambiguous relationship.

**Table 1:** The technological and morphological features of the analysed sickle elements from the site of Jeftelik.

Study number	Inventory number	Length mm	Width mm	Thickness mm	Blank	Backed blades	Cutting edge	Distal End	Proximal End
**1**	JTK08 DH906 UE36	26.1	30.6	7.8	blade	_	notch	broken	broken
**2**	JTK08 DF908 UE8	31.5	14.1	2	blade	_	notch	broken	broken
**3**	JTK08 DG906 UE24	48.1	20.9	8.3	blade	abrupt	_	truncation	broken
**4**	JTK08 DG907 UE24	45.1	21.2	8.1	blade	_	_	broken	broken
**5**	JTK09 K-L904-908 UE6B	20.8	7.8	2.7	bladelet	abrupt	_	broken	burin like scare
**6**	JTK10 DC910 UE10C	36	10.5	5	blade	abrupt	notch	truncation	broken
**7**	JTK10 DF909 UE22C-T2	34.2	10.2	3.3	bladelet	abrupt	covering retouch	broken	_
**8**	JTK10 DH908 US53	35.3	7.9	2.8	bladelet	helwan	_	_	_
**9**	JTK10 DJ910 UE22C N1764	31.1	6.5	2.6	bladelet	abrupt	inverse retouch, low angle	_	broken
**10**	JTK10 DJ913 UE22C-T2	24.2	12.8	3.8	bladelet	helwan	_	broken	broken
**11**	JTK10 DM908 UE22C-T2	19.5	9.3	3.7	bladelet	abrupt	_	broken	broken
**12**	JTK10 DP913 UE22C-T4	20.6	16.7	3.4	blade	semi-abrupt	_	truncation	broken

## Methodology

The analytical procedure consists of three-dimensional scanning of the glossed surfaces of both experimental and archaeological tools using confocal microscopy, followed by the measurement of surface texture through a series of quantitative parameters. These measurements are then used to generate a robust statistical model capable of discriminating between different experimental groups (wild cereals vs. other plants in this study). Finally, the model is applied to the archaeological tools in order to classify them within the experimental groups.

In this study we have applied a technical procedure previously used in other studies to identify cereal cutting in different states of maturity (domestic-ripe; wild cultivated-semi ripe; wild in natural stands-green) and reeds [37], to which we added grass in a following paper [38]. However, in this study, because of the nature of the question we want to solve, to determine whether glossed tools were used for harvesting cereals or other types of plants, the experimental program was divided into two groups: wild cereal-harvesting experiments reaped in natural stands and other plant-cutting experiments.

In this study, we also employ a new experimental procedure intended to identify the intensity of tool use, implemented by N. Mazzucco and J.J. Ibáñez [48], as a development of previous work focused on the analysis of the evolution of use-wear texture through the time of use [33]. Stands of domesticated wheat were harvested over a period of 36 hours. Every two hours, the edge of the sickle was cleaned and a silicone imprint was taken from the edge. This procedure allows us to build a model of gloss evolution over time, which can then be compared with the archaeological tools in order to estimate their duration of use.

The experimental model was developed using cereal-harvesting experiments and was therefore applied only to archaeological tools for which the classification model identified cereal harvesting as the dominant activity over the cutting of other plants. It should also be noted that the estimation of tool use duration is necessarily approximate, as the experimental reference collection differs from the archaeological assemblage in two important respects. First, the experimental program involved harvesting fully ripe domesticated cereals, whereas the archaeological tools were most likely used to harvest wild cereals growing in natural stands before full ripening. Second, the experimental gloss was measured on high-precision silicone replicas rather than directly on the flint surfaces, as was the case for the archaeological tools, which may introduce slight differences in surface texture measurements [49]. Despite these limitations, we consider the model sufficiently robust to provide a reliable approximation of tool use duration.

The predictive model of harvesting intensity was developed using a controlled experimental reference consisting of 36 hours of domestic wheat harvesting, during which silicone imprints of the use-wear traces were taken at two-hour intervals [48]. Harvesting intensity was operationalized as duration of use (in hours).

### Experimental reference framework

Archaeobotanical studies widely acknowledge that no domesticated cereals are documented in Natufian contexts [9,50]. Moreover, there is no indication of cereal cultivation during this period, particularly during the Early Natufian phase [51,52]. Consequently, harvesting experiments involving domesticated cereals or cultivated wild cereals were not included in the reference collection. The cereal-cutting experiments used in this study therefore consist exclusively of the harvesting of wild cereals in natural stands (Table 2).

**Table 2:** Details of the nine experimental sickle blades used for harvesting cereals, grasses, and reeds.

Tool	Type of plant	Plant species	Year	Place	Time of use	Sickles
**1**	Wild cereals	*Hordeum spontaneum* and *T. diccocoides*	2009	Jebel el Arab (Syria)	240′	One sickle (element A)
**2**	Wild cereals	*Hordeum spontaneum* and *T. diccocoides*	2009	Jebel el Arab (Syria)	240′	One sickle (element B)
**3**	Wild cereals	*Hordeum spontaneum* and *T. diccocoides*	2009	Jebel el Arab (Syria)	240′	One sickle (element C)
**4**	Wild cereals	*Hordeum spontaneum*	1993	Jordan Valley	120′	One sickle (element A)
**5**	Wild cereals	*Hordeum spontaneum*	1993	Jordan Valley	120′	One sickle (element B)
**6**	Wild cereals	*T. boeticum*	1995	Jebel el Arab (Syria)	720′	One sickle
**7**	Reeds	*Phragmites communis*	1993	Jalès (France)	100′	One sickle
**8**	Reeds	*Phragmites communis*	1993	Jalès (France)	120′	One sickle
**9**	Grass	*Ampelodesmos mauritanica*	2012	Ain Salem (Tunisia)	80′	One sickle

According to ethnographic observations, some plants (e.g., *Juncus*) are harvested by pulling them up rather than cutting them [53,54]. For this reason, the experimental program included two types of plants that are typically harvested with cutting tools: reeds (*Phragmites australis*) and feather grass (*Ampelodesmos mauritanica*).

The experiments involving the harvesting of wild cereals growing in natural stands were carried out in Jebel el Arab, southern Syria, in mid- and late May 1995 and 1996 by P. Anderson. Stands of *Triticum boeoticum* were harvested at a stage when most stems were beginning to yellow, and the first grains had detached from the seed head, but before full maturity. Sickles with wooden and antler handles were used for 11–13 hours, with fine-grained flint inserts. One element from this sickle was included in the experimental program.

In the same region, in 2009 and 2010, stands of *Triticum dicoccoides* and *Hordeum spontaneum* were harvested by J. J. Ibáñez for more than four hours at the beginning of June, when the grain had already formed but the plants were not yet fully ripe. A slightly curved sickle with a wooden handle was used, into which one obsidian and three fine-grained flint elements were inserted. Three elements from this sickle were included in the experimental program.

*Hordeum spontaneum* (wild barley) was also harvested using a bladed wooden sickle in 1993 in the Jordan Valley, for two hours by François Valla for P. Anderson. Two elements from this sickle were included in the experimental program. In all these experiments, wild cereal stems were held in groups in the hand and cut as close to the ground as possible, usually about 20 cm above the ground.

Reed-cutting experiments (*Phragmites australis*) were carried out in Jalès (France) by P. Anderson in shallow standing water, at a stage when the stems were firm but not brittle. Two blades used for cutting reeds in two separate experiments were included in the experimental program.

Grass-cutting experiments were conducted in Tunisia by P. Anderson [54]. *Ampelodesmos mauritanica* is a fibrous grass belonging to the Stipeae tribe, known as *dis* or *diss* in Arabic. It is harvested with sickles and used as fodder, as well as for making baskets, mats, ropes, and whips, and for roofing huts [55]. One element from this cutting tool was included in the experimental program.

The experiment for building the model of harvesting intensity was conducted in north-eastern Tuscany (Italy) by N. Mazzucco. Two experimental plots cultivated with *Triticum aestivum*’, through organic and low-input farming practices. Cereal was harvested with an experimental sickle realized on *Quercus* sp., taking a complete sickle from the La Marmotta Early Neolithic site as a reference model [56]. For each field, approximately 400 m^2^ were harvested over three working days. For the sickle used as the reference in this study, harvesting totalled 36 hours of work in mid-June —12 hours for three seasons (2019, 2021, 2022). The cereal was in the hard-dough stage of ripening. the tool. To model the progression of use-wear through time, moulds of the lithic surfaces were taken every two hours of use, from 0 h to 36 h, using a hydrophilic polyvinyl siloxane impression material (Light Fast Set, Heraeus Kulzer). Before moulding, artefact surfaces were carefully cleaned with distilled water followed by ethanol. A total of 19 impressions were produced (0 h, 2 h, 4 h, 6 h, 8 h, 10 h, 12 h, 14 h, 16 h, 18 h, 20 h, 22 h, 24 h, 26 h, 28 h, 30 h, 32 h, 34 h, 36 h). Impressions from on the inserts of the experimental sickle were used to build the wear evolution model [48].

### Technical procedure

The experimental and archaeological tools were washed using soapy water, with the surfaces gently rubbed by hand, and subsequently dried with a hand dryer to eliminate any remaining moisture. Surface areas measuring 650 × 500 μm were captured using a Sensofar Plu Neox white light scanning confocal microscope equipped with a 20 × objective (NA 0.45), enabling high-resolution documentation of surface texture. The spatial sampling interval was set to 0.83 μm, with an optical resolution of 0.31 μm, a vertical resolution of 20 nm, and a z-step of 0.5 μm.

Acquisitions were taken along the edges of tools, focusing on areas exhibiting the most developed use--wear polish associated with plant harvesting in order to maximize the reliability of functional discrimination. The 3D images of 650 × 500 μm acquired with the confocal microscope were processed using the software MountainsMap (v.8) from Digital Surf to extract 3D texture parameters. From each acquisition, between one and four subsamples of 200 × 200 μm were extracted (Table 3), which corresponds to the standard size used in our previous methodology to discriminate plant-related cutting polishes [34,36–39,43,57].

**Table 3:** Number of measurements obtained for each tool.

N tool	N measurements per tool
**1**	21
**2**	29
**3**	21
**4**	21
**5**	36
**6**	31
**7**	33
**8**	35
**9**	31
**10**	31
**11**	28
**12**	23
**Total**	340

Three ensembles of 3D surfaces (200 × 200 μm) were analysed: 171 surfaces (dataset in S1 Table) from nine experimental tools used to identify the type of harvested plants (either wild cereals or other plants); 264 surfaces (dataset in S1 Table) from 18 experimental sickle groups used to model the intensity of use; and 340 surfaces (dataset in S1 Table) (Figures in S1 Fig) from twelve archaeological tools from Jeftelik.

## Results

### Harvesting regime

Quadratic Discriminant Analysis (QDA) was applied to the dataset (S1 Table) to evaluate the multivariate separability of experimental groups of wild cereal (Group 1) and other plants (reeds + grass) (Group 2) harvesting. To identify the most relevant discriminant 3D texture parameters, a stepwise procedure using Wilks’ lambda distribution test was performed, choosing MeanDepthFurrow, Sa, Sz, and Sal as textural predictor variables. Prior probabilities were set equal (0.50 per group) to avoid classification bias due to unequal sample sizes. Box’s M test indicated significant differences between group covariance matrices (M = 18.626, F = 18.506, p < 0.001), violating the homoscedasticity assumption required for Linear Discriminant Analysis and justifying the use of QDA, which accommodates group-specific variance–covariance structures.

The discriminant function was statistically significant (Wilks’ λ = 0.528, χ^2^(4) = 106.80, p < 0.001), confirming multivariate differences between groups. The eigenvalue (0.896) and canonical correlation (r = 0.687) indicate substantial discriminatory power, with approximately 47% of the variance in group membership explained (r^2^ = 0.472). Overall classification accuracy reached 84.8%, with 88.4% of Group 1 and 78.0% of Group 2 correctly classified, and misclassification rates of 11.6% and 22.0%, respectively (Table 4).

**Table 4:** The classification results, for two ensembles: nine experimental tools used to identify the type of harvested plants, and twelve archaeological tools from Jeftelik, 84,8% of original grouped cases were correctly classified.

Original		Predicted classification		
		Wild cereals	Reeds and grasses	Total
**Count**	Wild cereals **(experimental tools)**	99	13	112
	Reeds and grasses **(experimental tools)**	13	46	59
	Archaeological tools	212	128	340
**%**	Wild cereals **(experimental tools)**	88.4	11.6	100.0
	Reeds and grasses **(experimental tools)**	22.0	78.0	100.0
	Archaeological tools	62.4	37.6	100.0

To assess potential overfitting, a leave-one-out cross-validation (LOOCV) procedure was performed on the experimental dataset. Cross-validated classification rates remained comparable, with 87.5% correct classification for the wild cereal cutting group and 78.0% for the reeds and grasses group, indicating stable and robust model performance.

The model was subsequently applied to 12 archaeological tools, comprising 340 ungrouped cases corresponding to multiple 200 × 200 µm surface samples measured for each artefact. Of these sampled surfaces, 62.4% were classified as belonging to the group associated with cutting wild cereals in natural stands, whereas 37.6% were assigned to the group associated with cutting other plants (Table 5). Because the number of sampled surfaces varied between tools, proportions were recalculated after equalizing the number of samples per tool. This normalization produced similar results, with 61.1% assigned to cereals and 38.9% to other plants.

**Table 5:** The functional classification and estimated use duration of the archaeological tools.

Study number	Inventory number	% Wild cereals	% Reeds and grasses	Interpretation	Hours of use
**1**	JKT08 DH906 UE36	61.9	38.1	Mixed plants	
**2**	JTK08 DF 908 UE8	58.6	41.4	Mixed plants	
**3**	JTK08 DG906 UE24	61.9	38.1	Mixed plants	
**4**	JTK08 DG907 UE24	81	19	Wild cereals	20.5
**5**	JTK09 K-L904-908 UE6B	77.8	22.2	Wild cereals	23.8
**6**	JTK10 DC910 UE10C	83.9	16.1	Wild cereals	18.7
**7**	JTK10 DF909 UE22C-T2	75.8	24.2	Wild cereals	24.9
**8**	JTK10 DH908 US53	74.3	25.7	Wild cereals	13.1
**9**	JTK10 DJ910 UE22C N1764	41.9	58.1	Mixed plants	
**10**	JTK10 DJ913 UE22C-T2	51.6	48.4	Mixed plants	
**11**	JTK10 DM908 UE22C-T2	60.7	39.3	Mixed plants	
**12**	JTK10 DP913 UE22C-T4	4.3	95.7	Reeds and grasses	

Tool-by-tool classification of the surface samples indicates a predominant use in wild cereal harvesting for five tools (4, 5, 6, 7, and 8) (Figs 3 and 4), all of which show more than 74% of their samples assigned to the wild cereals group. In contrast, Tool 12 exhibits a clear predominance of reeds and grasses (95.7%) (Fig 5), indicating primary use in cutting other plants.

**Fig 3:**
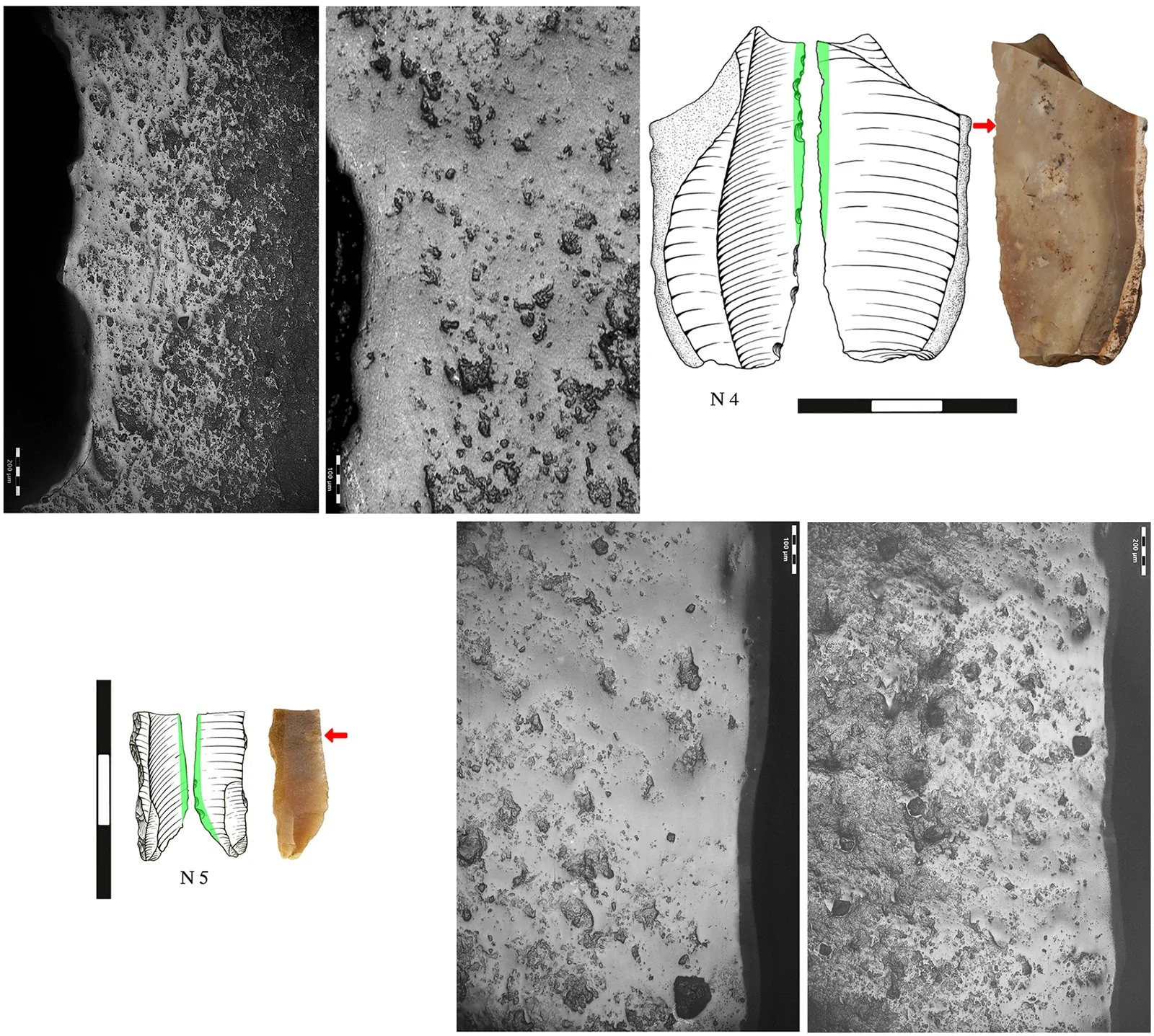
Tools Nos. 4 (JTK08 DG907 UE24) and 5 (JTK09 K-L904-908 UE6B) from the Jeftelik site (Early Natufian) are classified as wild cereal harvesting tools. Micrographs were taken with a CCD (charge-coupled device) camera attached to a Olympus BHM reflected-light (metallographic) microscope.

**Fig 4:**
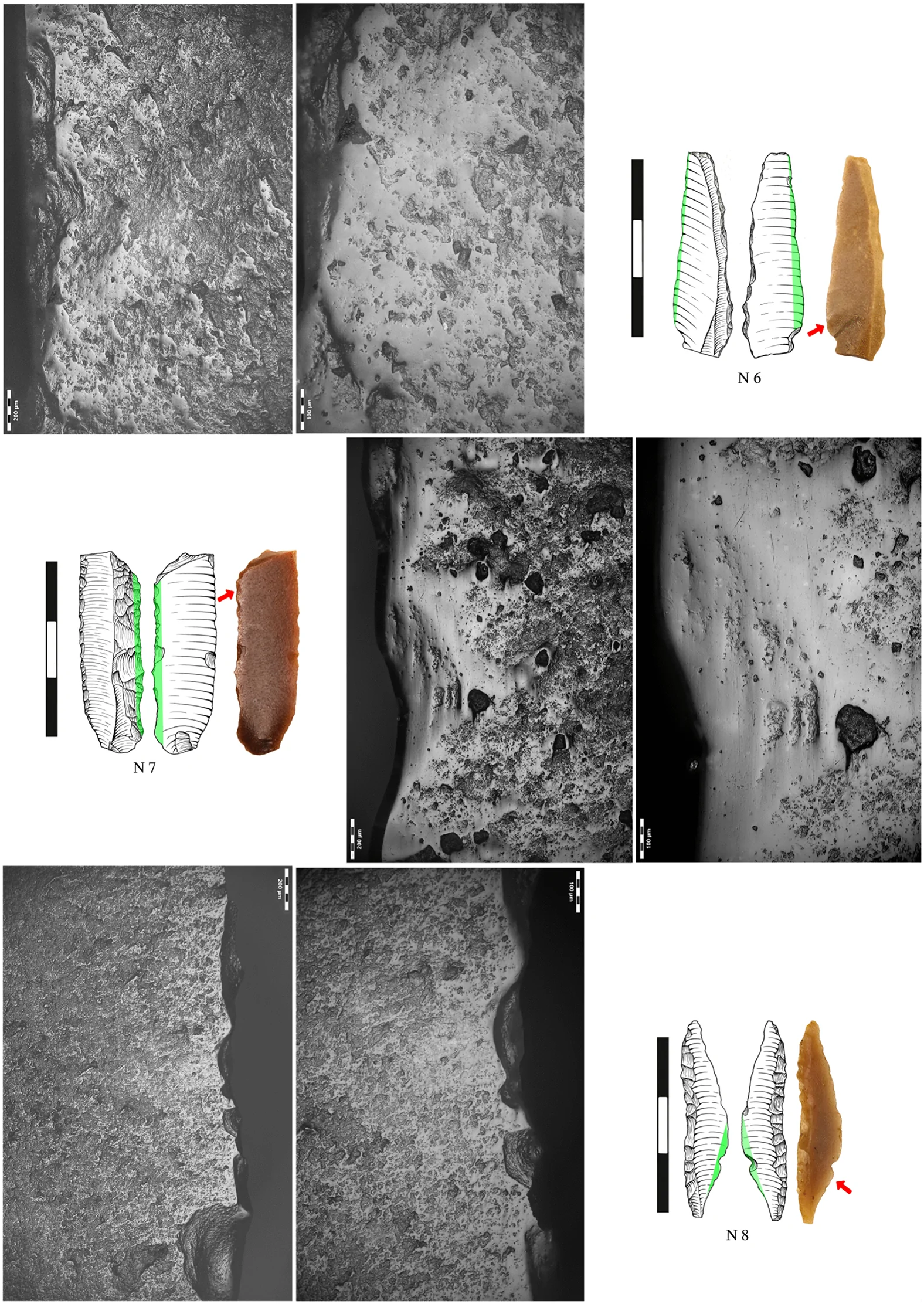
Tools Nos. 6 (JTK10 DC910 UE10C), 7 (JTK10 DF909 UE22C-T2) and 8 (JTK10 DH908 US53) from the Jeftelik site (Early Natufian) are classified as wild cereal harvesting tools. Micrographs were taken with a CCD (charge-coupled device) camera attached to a Olympus BHM reflected-light (metallographic) microscope.

**Fig 5:**
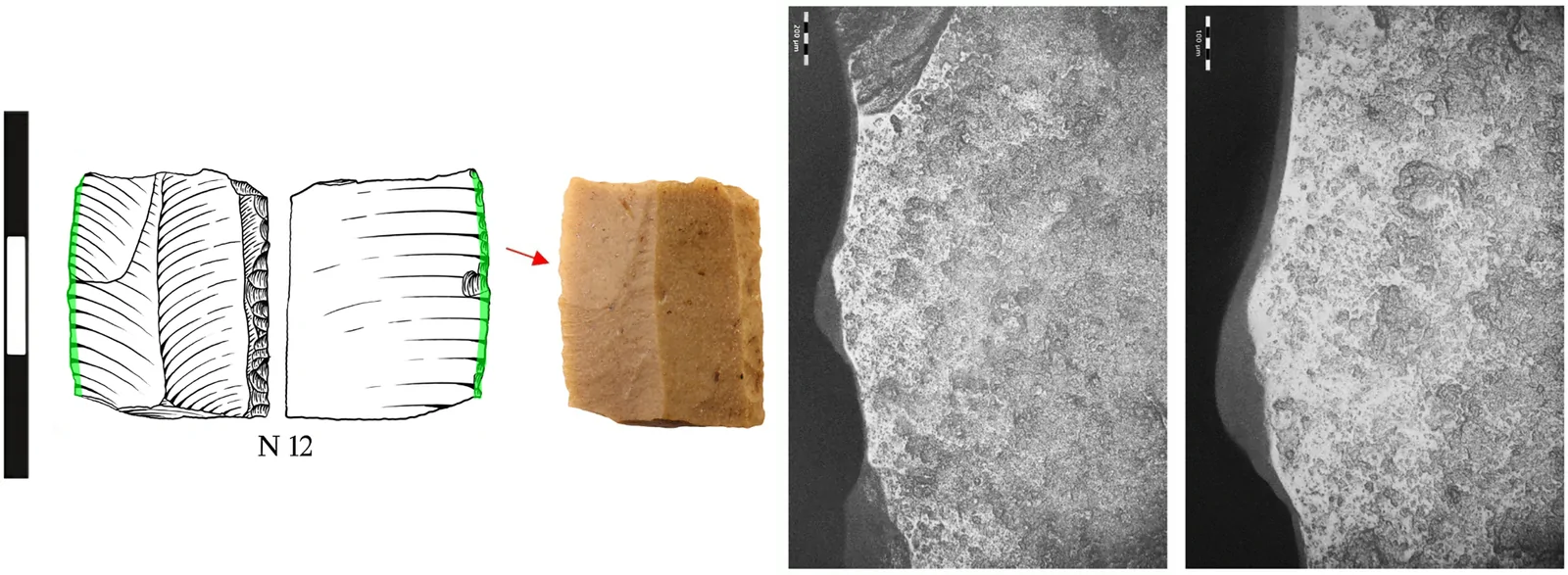
Tool No. 12 (JTK10 DP913 UE22C-T4), from the Jeftelik site (Early Natufian) is classified as reed harvesting tool. Micrographs were taken with a CCD (charge-coupled device) camera attached to a Olympus BHM reflected-light (metallographic) microscope.

The remaining six tools (1, 2, 3, 9, 10, and 11), display more balanced proportions, suggesting mixed plant processing activities (Fig 6 and 7). Although wild cereal cutting predominates in Tools 1, 3, and 11 (approximately 60–62%), a substantial proportion of samples is assigned to other plants, indicating that cereals were important but not exclusive targets. Tools 9 and 10 show near-equivalent distributions between the two categories, reinforcing the interpretation of polyvalent use (Table 5).

**Fig 6:**
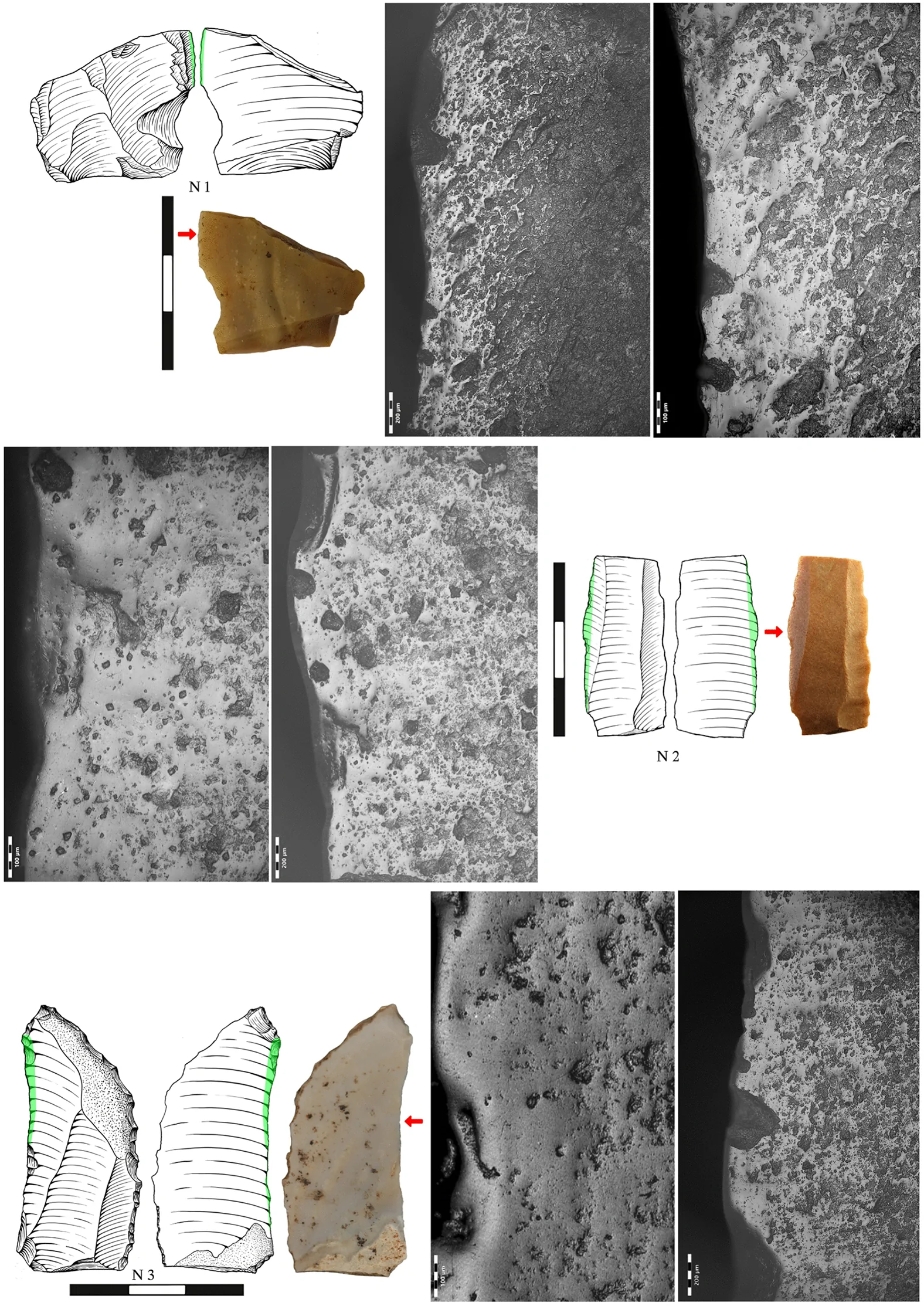
Tools Nos. 1 (JTK08 DH906 UE36), 2 (JTK08 DF908 UE8) and 3 (JTK08 DG906 UE24) from the Jeftelik site (Early Natufian) are classified as mixed plant harvesting tools. Micrographs were taken with a CCD (charge-coupled device) camera attached to a Olympus BHM reflected-light (metallographic) microscope.

**Fig 7:**
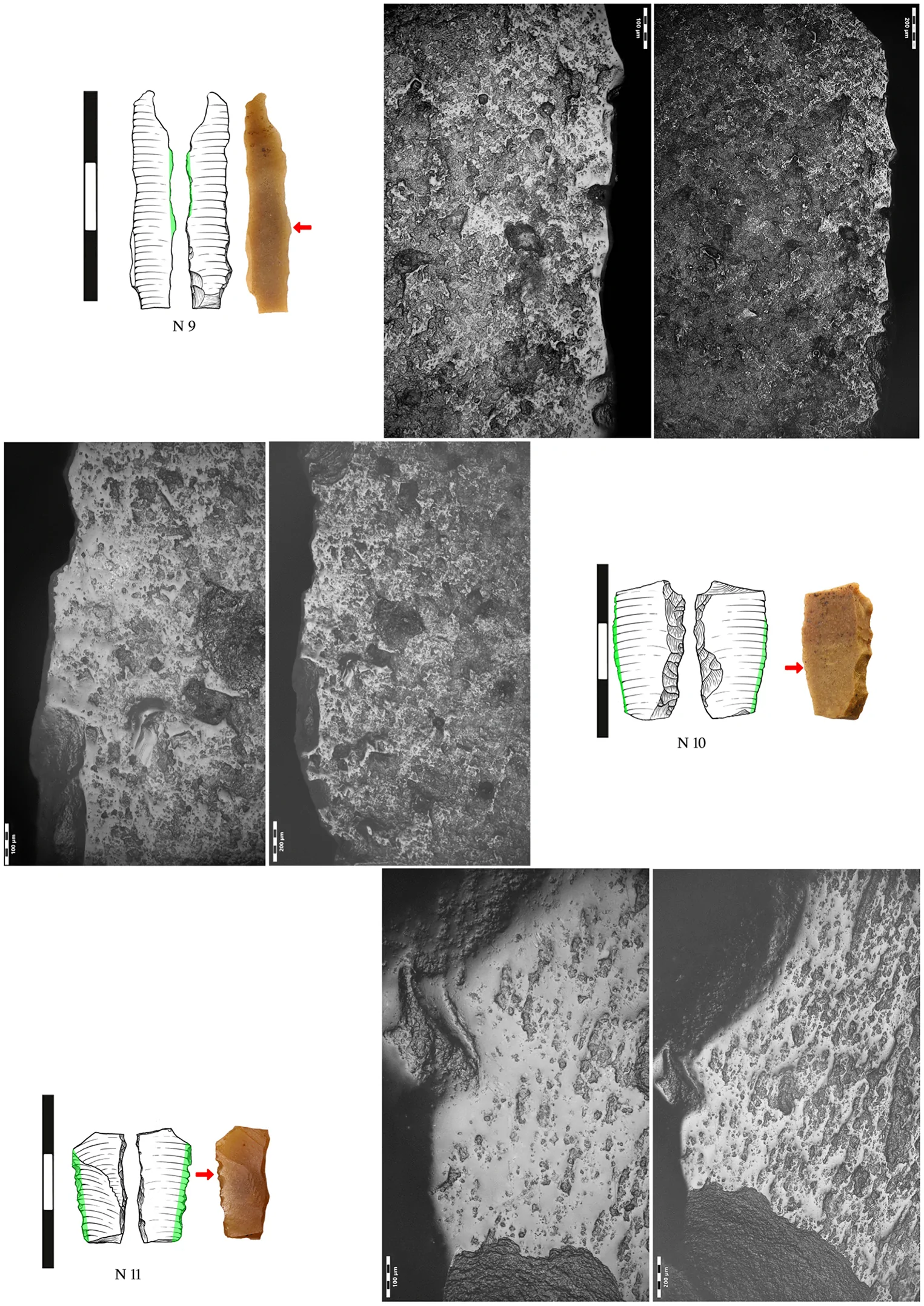
Tools Nos. 9 (JTK10 DJ910 UE22C N1764), 10 (JTK10 DJ913 UE22C-T2) and 11 (JTK10 DM908 UE22C-T2) from the Jeftelik site (Early Natufian) are classified as mixed plant harvesting tools. Micrographs were taken with a CCD (charge-coupled device) camera attached to a Olympus BHM reflected-light (metallographic) microscope.

### Harvesting intensity

To estimate the intensity of use of cereal harvesting tools, a predictive model was developed using Random Forest Regression, trained on the experimental dataset (S1 Table), in which each analysed surface is associated with a controlled duration of use expressed in hours. The analyses were performed using a Python-based computational environment.

The target variable was use duration (in hours). This variable represents a direct proxy for harvesting intensity and enables the implementation of a supervised learning approach capable of predicting use intensity from surface texture parameters.

Missing predictor values were handled by median imputation. No variable scaling was applied, as tree-based algorithms are insensitive to monotonic transformations of the predictors.

The model was implemented using a Random Forest Regressor with 500 trees (random_state = 42). This algorithm is particularly suitable for this type of data because it can capture non-linear relationships and complex interactions among highly correlated variables, as commonly observed in surface texture datasets. The model was evaluated using 5-fold cross-validation. Mean performance across folds was R^2^ = 0.803, MAE = 3.45 h and RMSE = 4.74 h. Out-of-fold prediction performance was R^2^ = 0.807, MAE = 3.45 h and RMSE = 4.80 h. The model shows high predictive performance (R^2^ ≈ 0.80–0.83), indicating a strong relationship between surface texture parameters and use duration, with prediction errors typically within ±3–5 hours.

Once validated, the model was applied to the archaeological dataset (S1 Table), generating predicted use durations for each analysed surface. For interpretative purposes, predicted values were classified into four intensity categories defined by the quartiles of the experimental use-duration distribution: G1 (Low), ≤ 8 h; G2 (Medium), > 8 h to 18 h; G3 (High), > 18 h to 28 h; and G4 (Very high), > 28 h. This classification framework enables direct comparison between archaeological samples and experimentally controlled reference data. For each tool, all surface predictions were averaged, and the mean value was then assigned to one of the four intensity groups (Table 5). The experimental model for estimating use intensity was developed using cereal-harvesting experiments. Therefore, it was applied only to the five archaeological tools for which the classification model indicated that wild cereal harvesting was the dominant activity.

Application of the model to the archaeological assemblage indicates that two tools exhibit intermediate use, between 10 and 20 hours (6 and 8), whereas three tools show evidence of more prolonged use, for more than 20 hours (4, 5 and 7) (Table 6). This analysis reveals that approximately two-thirds to 90% of the surfaces fall within a single use-intensity category. Specifically, while Tools 5 and 7 show highly homogeneous classifications, the other three tools display greater variability, indicating that, in some cases, use intensity varies along the cutting edge (Table 6).

**Table 6:** Shows the classification of individual surfaces for each tool.

Tool	≤8 h	>8–18 h	>18–28 h	>28 h	Total surfaces	Mean use duration (h)
**4**	0 (0.0%)	6 (28.6%)	14 (66.7%)	1 (4.8%)	21	**20.47**
**5**	0 (0.0%)	2 (5.6%)	33 (91.7%)	1 (2.8%)	36	**23.82**
**6**	4 (12.9%)	7 (22.6%)	20 (64.5%)	0 (0.0%)	31	**18.66**
**7**	0 (0.0%)	2 (6.1%)	27 (81.8%)	4 (12.1%)	33	**24.94**
**8**	6 (17.1%)	23 (65.7%)	5 (14.3%)	1 (2.9%)	35	**13.15**

## Discussion

Our study demonstrates that wild cereals were harvested at the Early Natufian site of Jeftelik approximately 14,000 years ago. Bladelets were mounted into straight composite sickles and used to cut plants, predominantly cereals. The tools used for cutting wild cereals were employed over considerable periods, three of them exceeding 20 hours.

However, although cereal harvesting with cutting tools could be relatively intensive, the overall scale of sickle-based harvesting within the lithic assemblage at Jeftelik appears limited. Glossed tools represent only a minor component of the assemblage: approximately 1% of retouched tools display macroscopic gloss. When compared with lunates—characteristic Natufian tools whose function as projectile elements is well established—the proportion of glossed tools amounts to approximately 1:14. In five tools, cereal cutting clearly predominates, whereas six tools show mixed use, indicating that they were employed to cut not only cereals but also other plants. This suggests that a substantial proportion of plant-cutting implements were not exclusively dedicated to cereal harvesting but were used more generally as multipurpose plant-cutting tools. Together, these data indicate that cereal cutting was practiced, but not at a scale implying systematic wild cereal collection using a reaping technique.

This limited representation of cereal-harvesting tools appears to reflect a long-term behavioural pattern. At Ohalo II (ca. 23,000 BP), five plant-cutting tools were identified (0.1% of retouched tools) within a toolkit comprising approximately 100,000 artefacts, and the microwear characteristics of at least one specimen suggest wild cereal harvesting [40]. Similarly, small numbers of glossed tools occur at Epipalaeolithic sites predating the Natufian, such as Ein Gev I [58].

During the Natufian period, the quantity of glossed tools remains limited (Table 7). Most sites show proportions similar to or lower than those observed at Jeftelik. Only a few sites display slightly higher values, ranging between 2.5% and 5%, such as Dederiyeh, Hayonim Cave, Nahal Oren, and Ein Gev II (Table 7). In terms of their relative importance compared to lunates—tools associated with hunting activities—the ratio of glossed tools varies across sites. In some cases (e.g., Hayonim Cave IV–V, Mallaha IB, Nahal Oren, Wadi el-Hemmeh 27, and Ein Gev II), ratios range between approximately 1:1.3 and 1:5.2, whereas in others (e.g., Abu Hureyra, Dederiyeh Cave, Mureybet IA, Qarassa 3, Jeftelik, El-Wad, and Hayonim Terrace), they are considerably lower, ranging from 1:8–1:27.

**Table 7:** The proportions of sickle blades and lunates across various Natufian sites in the Near East.

Region	Sites	Period	Date cal. BP	% of Sickle blades	% of Lunates	Sickle Blades / Lunates	Reference
**North Levant**	**Abu Hureyra (1)**	Late Natufian	12650	0.39%	5.7%	1/14.6	[59]
	**Dederiyeh Cave**	Early and Late Natufian	14.500-12.700	4.09%	32.6%	1/8	[60]
	Mureybet (Ia)	Late Natufian	11950	1.2%	13.9%	1/11.5	[61]
	**Qarassa 3**	Early Natufian	13.385	0.2%	4.7%	1/23.5	[46], and the Study of Khaled Abdo
	**Jeftelik**	Early Natufian	14.190-13.830	1%	14.7%	1/13.7	[45], and the excavation Report
**South Levant**	**El-Wad**	Late Natufian	?	1.2%	11.15%	1/9.3	[62]
	**Hayonim Terrace**	Late Natufian	13.300–12.100	0.6-1.0%	22.1%	1/27.6	[22,63]
	**Hayonim Cave IV-V**	late Natufian	12.528	2.7-3.1%	14.2-14.5%	1/5.2 1/4.6	[63]
	**Mallaha IB**	Final Natufian	12.140-11.750	1,91%	7.52%	1/3.9	[64]
	**Nahal Oren (Natufian phase)**	Late Natufian	13.050-11.650	3.5%	4.8%	1/1.3	[65]
	**Wadi el-Hammeh 27**	Early Natufian	14.500–14.000	2.5%	12.3%	1/4.9	[66]
	**Ein Giv II**	Late Natufian	12.600 - 12.050	3.3%	4.1%	1/1,24	[67]

The small number of glossed tools could suggest that wild cereals were exploited only sporadically, perhaps during periods of resource scarcity. However, this explanation is unlikely. The exploitation of cereals requires relatively complex toolkits and technical processes, including dehusking, storage, and pounding or milling, which are difficult to maintain for a purely sporadic resource. At Jeftelik, Natufian levels yielded seven fragments of grinding slabs and two handstones. In addition, surface surveys identified mortars, pestles, and grinding slabs that, based on their typology, likely correspond to Natufian occupations (Fig 8). These findings indicate that the technological system for processing plant resources was present, even if the number of cereal-reaping tools was limited.

**Fig 8:**
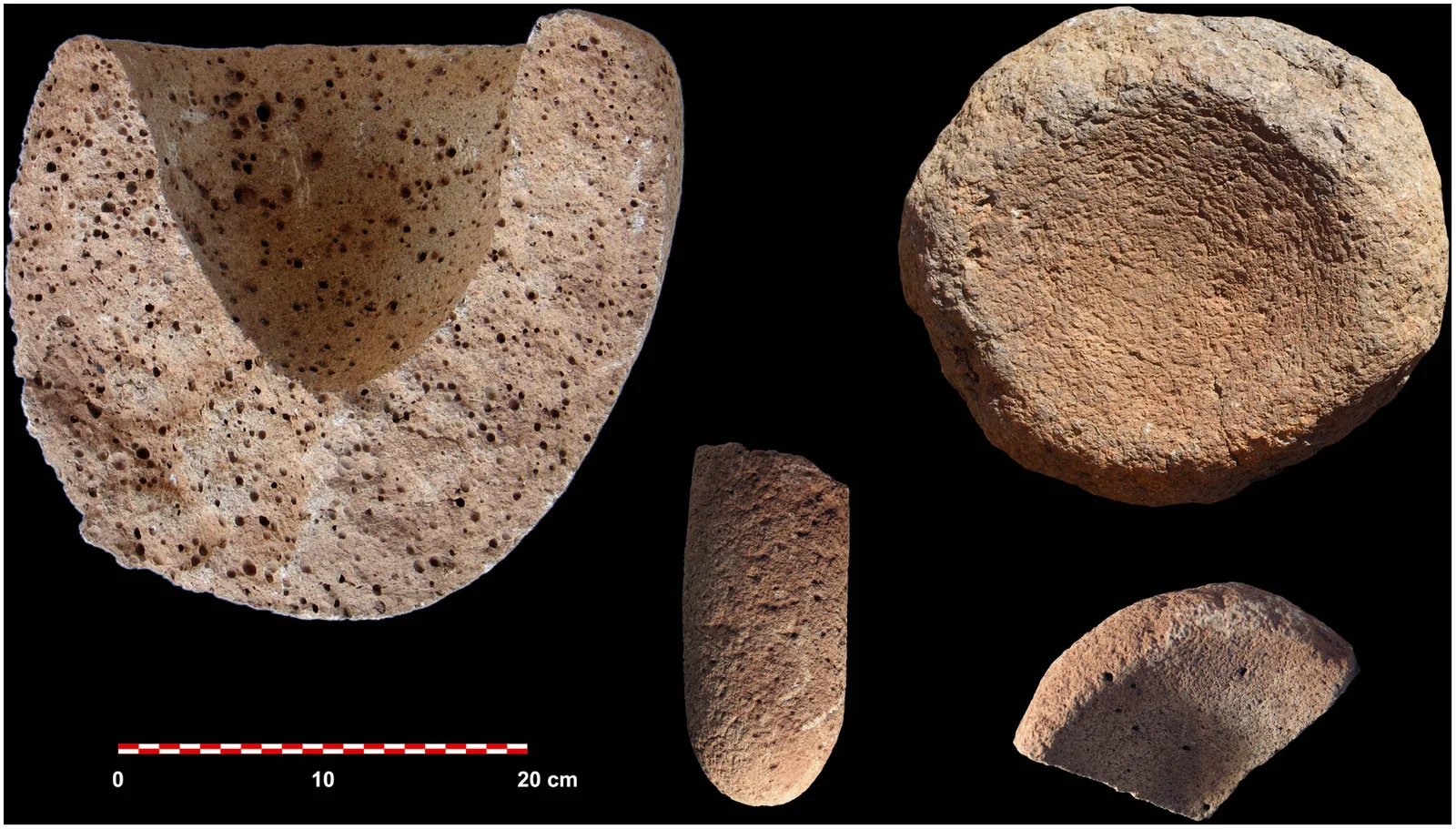
Ground stone tools from Jeftelik: (a) mortar; (b) pestle; (c, d) grinding slabs.

The site of Qarassa 3 provides a comparable example of a limited number of harvesting tools associated with a complex cereal-processing system. Excavations revealed portions of three dwellings among twelve structures arranged in an arc [18]. More than eighty deep mortars and several cupholes were documented in the basaltic surface near the dwellings. Use-wear and residue analyses indicate that at least some of these mortars were used for cereal processing, likely involving pounding within the rock using wooden implements for dehusking. However, only two glossed tools (0.2%) were identified at the site. Similarly, ground stone toolkits are well represented at other Natufian sites [68,69] and are widely interpreted as evidence for cereal processing [18,70], despite the limited presence of cereal-cutting tools.

Taken together, the evidence indicates that wild cereals were indeed exploited during the Natufian period, but that sickle harvesting constituted only a minor component of a broader and more flexible plant-procurement system. Ethnographic and experimental studies demonstrate that wild cereals can be collected using a variety of techniques, including hand-plucking, uprooting, and beating seed heads into containers, in addition to cutting with sickles [71,72]. This diversity of harvesting strategies may explain why sickle blades remain relatively rare in many Epipalaeolithic and Natufian assemblages despite clear evidence of cereal exploitation.

If cereals were frequently collected using methods such as hand-plucking or uprooting, sickle harvesting likely represented only one of several complementary strategies. This raises the question of why stem cutting with sickles was practiced at all if other methods were more common. One possible explanation is that sickle harvesting represents a strategy aimed at maximizing yield and efficiency, reflecting episodes of intensified plant exploitation. Sickle use allows large quantities of stems to be cut rapidly, which may have been particularly advantageous during short harvesting windows when cereals reached peak ripeness [73,74]. Wild cereal productivity varies markedly between years as a function of temperature and precipitation [75,76] We hypothesize that sickle harvesting would have been particularly advantageous in years when wild cereals growing in natural stands were especially abundant, making more efficient harvesting methods necessary to maximize yields.

The relative rarity of sickle blades is therefore unlikely to reflect the marginal importance of cereals. Rather, it probably reflects the diversity of harvesting techniques employed—including uprooting, ear-plucking, and sickle harvesting—among which cutting with sickles represented a specialized strategy used when rapid and efficient harvesting of large quantities of cereals was required.

## Conclusions

This study provides quantitative and functional evidence for the harvesting of wild cereals at the Early Natufian site of Jeftelik, contributing to a more nuanced understanding of plant exploitation strategies during the Late Epipalaeolithic. Through the combined use of confocal microscopy, three-dimensional surface texture analysis, and supervised classification and regression methods—including Quadratic Discriminant Analysis (QDA) and Random Forest—it has been possible to reconstruct both the function and intensity of use of glossed lithic tools with a high degree of confidence.

The results indicate that wild cereal harvesting was the primary function of glossed tools, although other siliceous plants, such as reeds and grasses, were also processed. At the same time, use-intensity estimates for wild cereal harvesting tools reveal a relatively intensive use, with three of the five tools showing more that 20 hours of work.

Despite clear evidence for cereal harvesting during relatively long periods of work, glossed sickle blades represent only a very small proportion of the lithic assemblage at Jeftelik. When considered together with the presence of a developed ground stone toolkit—indicating the processing of cereals and other plant resources—these results suggest that wild cereals formed an important component of subsistence practices, but that sickle harvesting was not the dominant method of their procurement.

Instead, the evidence supports a flexible and diversified plant-procurement system, in which multiple harvesting techniques were employed. The limited number of glossed tools and the frequent presence of mixed plant-cutting signals indicate that sickles were not routinely used for cereal collection. Similar patterns observed at other Epipalaeolithic and Natufian sites reinforce this interpretation.

Within this framework, sickle harvesting may be better understood as a strategy of situational intensification, employed under specific conditions when maximizing the yield of ripening cereal stands was advantageous. In such contexts, sickles would have enabled the rapid and efficient harvesting of large quantities of plants within a limited time window. Outside these episodes, cereals were likely collected using a broader range of techniques, including hand-plucking, uprooting, or other low-investment methods.

More broadly, these findings highlight the importance of integrating quantitative modelling with functional analysis to reconstruct past behaviours. The approach developed here demonstrates that surface texture analysis, combined with machine learning techniques, can provide robust estimates of tool use intensity and offer new insights into the organisation of subsistence practices. In this perspective, the Natufian exploitation of wild cereals appears not as a simple precursor to agriculture, but as part of a complex, flexible, and context-dependent system of resource management.

## Supporting information

S1 Fig Mnt File Confocal microscopy raw image montage file. (MNT)

S1 Fig Supplementary figures containing confocal microscopy images. (PDF)

S1 Table Supplementary table containing measurement data. (XLSX)
