# Supplementary material for: Wild cereal harvesting ca. 14,000 years ago at Jeftelik (Western Syria)
Source: PLoS One. 2026 Nov 10;21(11):e0350364. doi: 10.1371/journal.pone.0350364 (PMC13750610; doi:10.1371/journal.pone.0350364)
Supplement: Supplementary file 1 [file pone.0350364.s002.pdf]

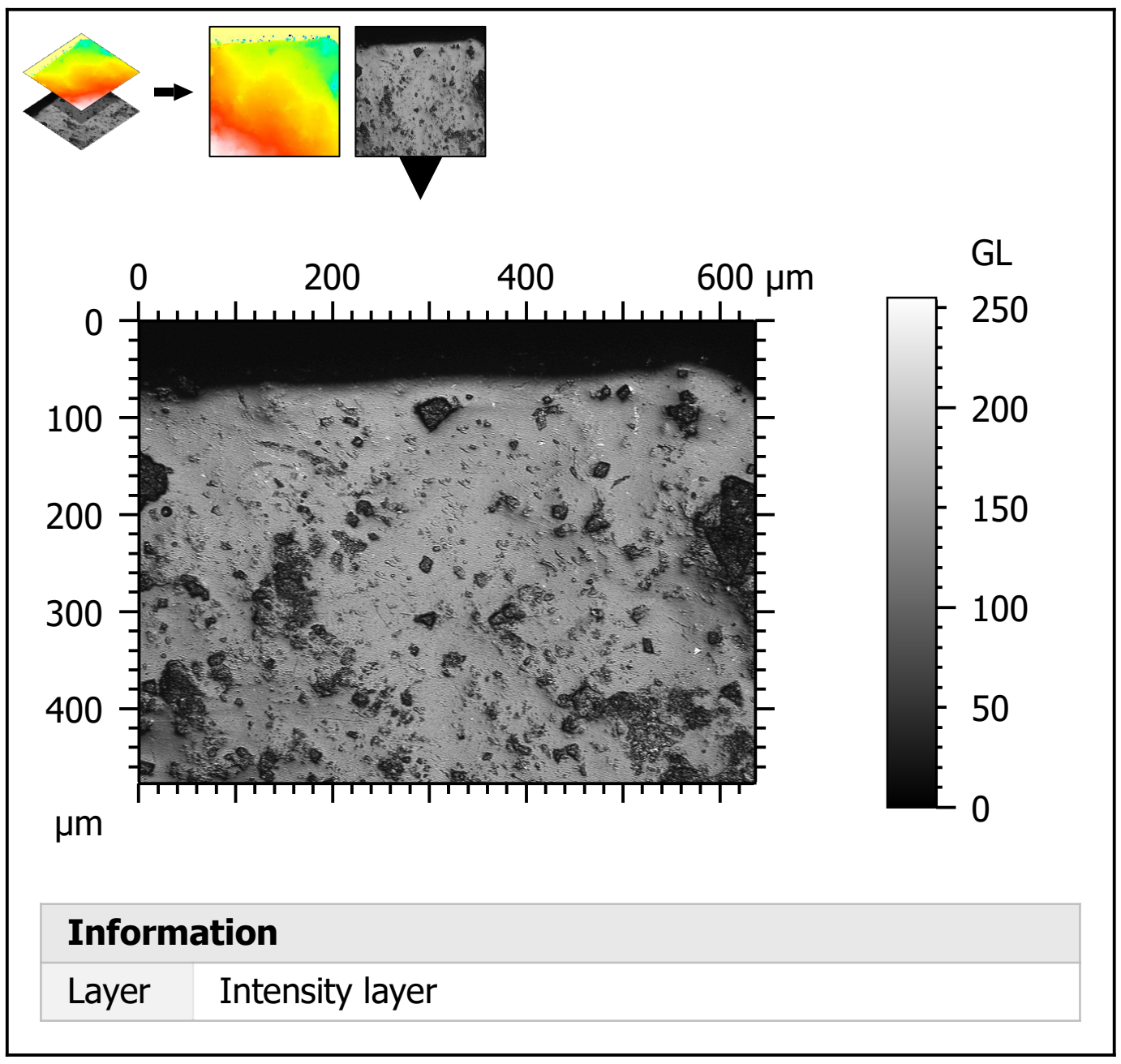

Scanned surface JTK08 DF 908 UE8 N 2 CD9-1, 650x450µ. Intensity image

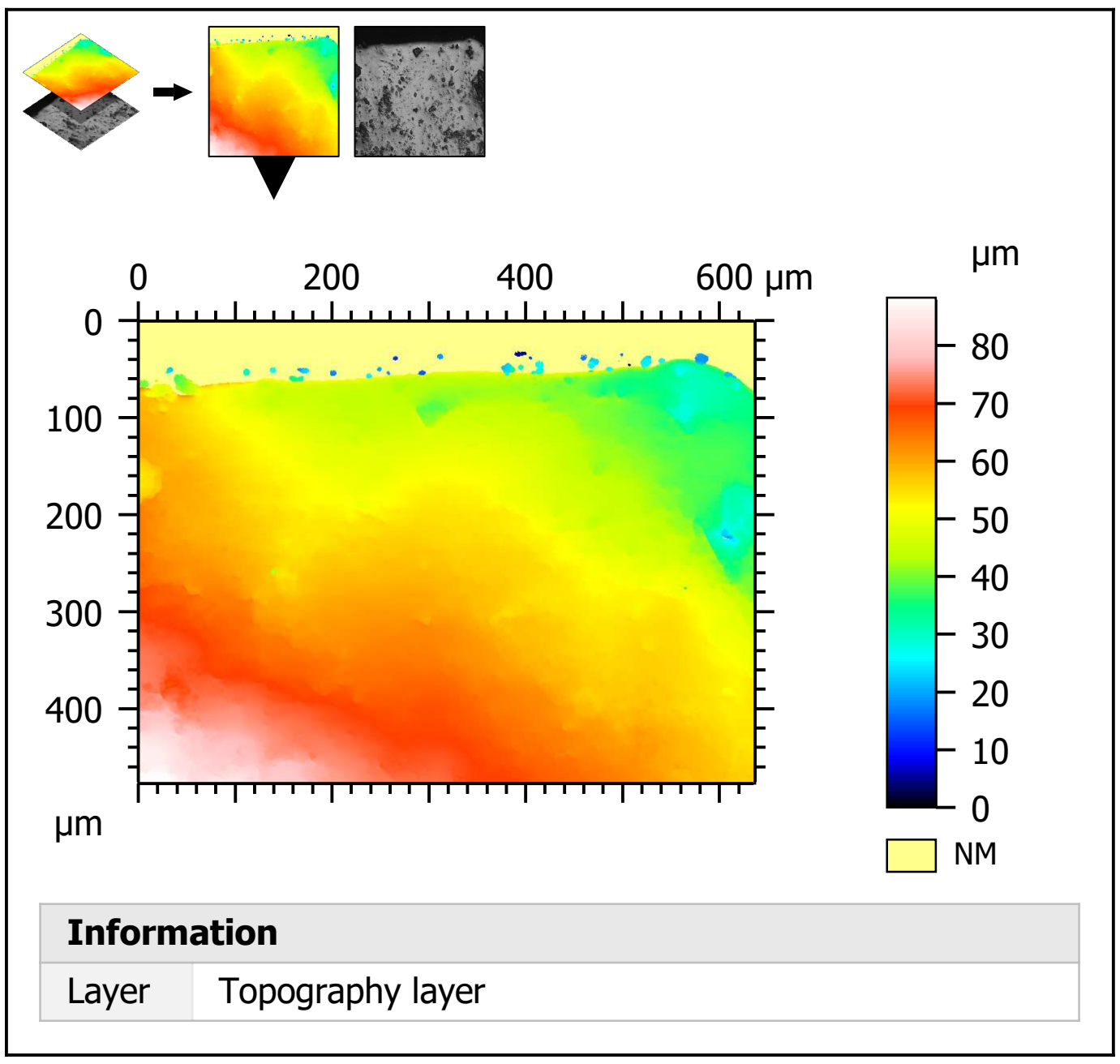

Scanned surface JTK08 DF 908 UE8 N 2 CD9-1, 650x450µ. Topography

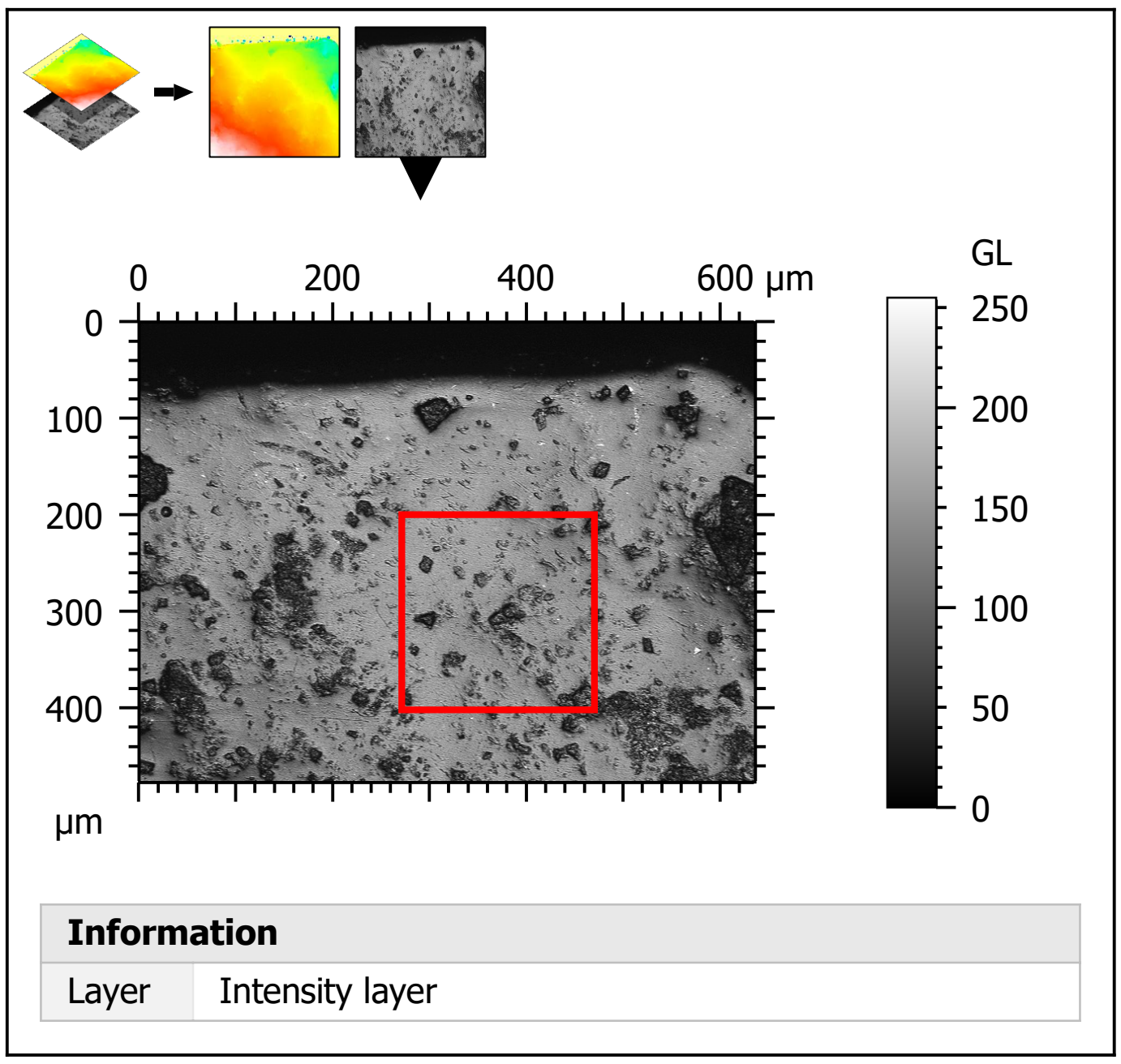

Square of 200x200µ to be sampled

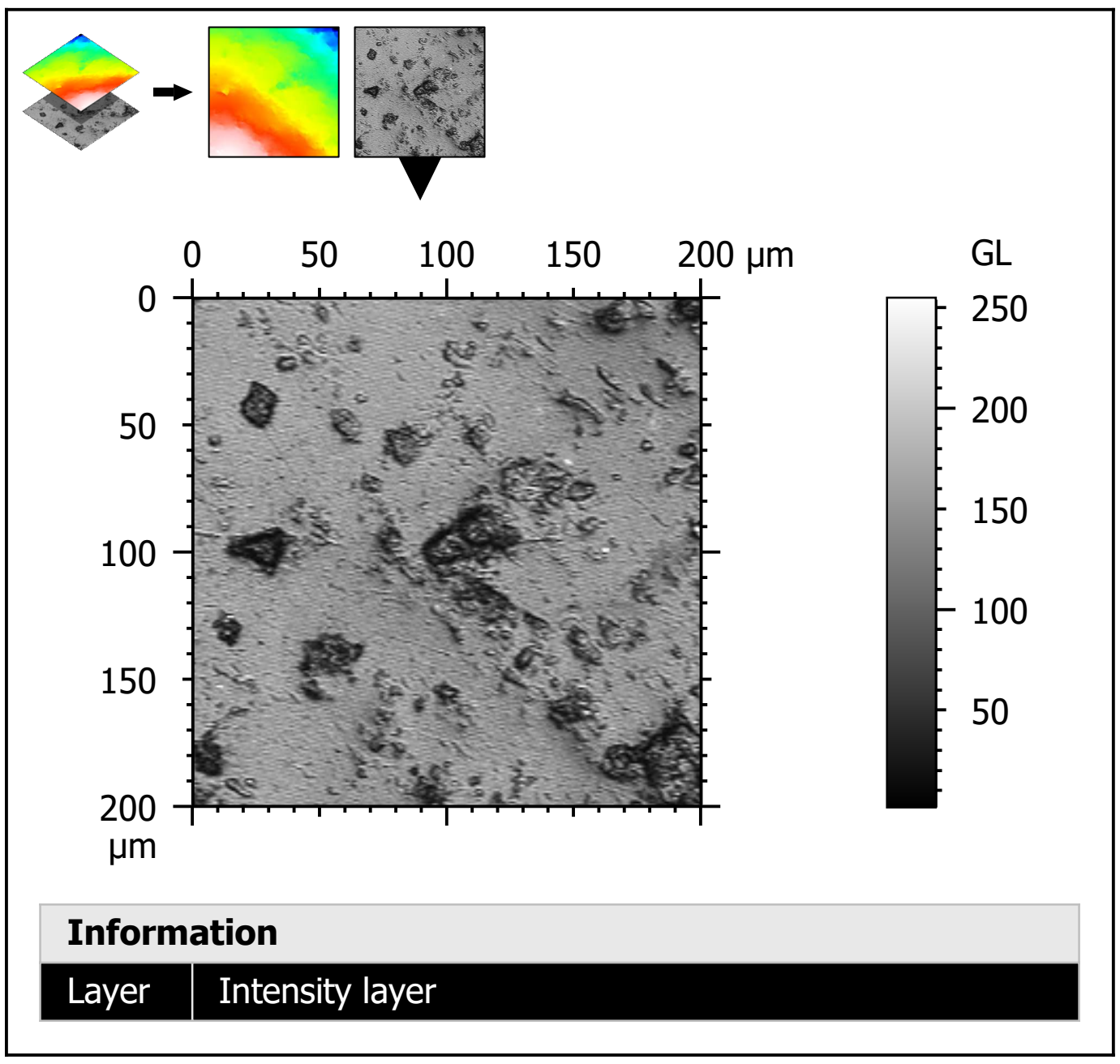

200x200µ sampled area,

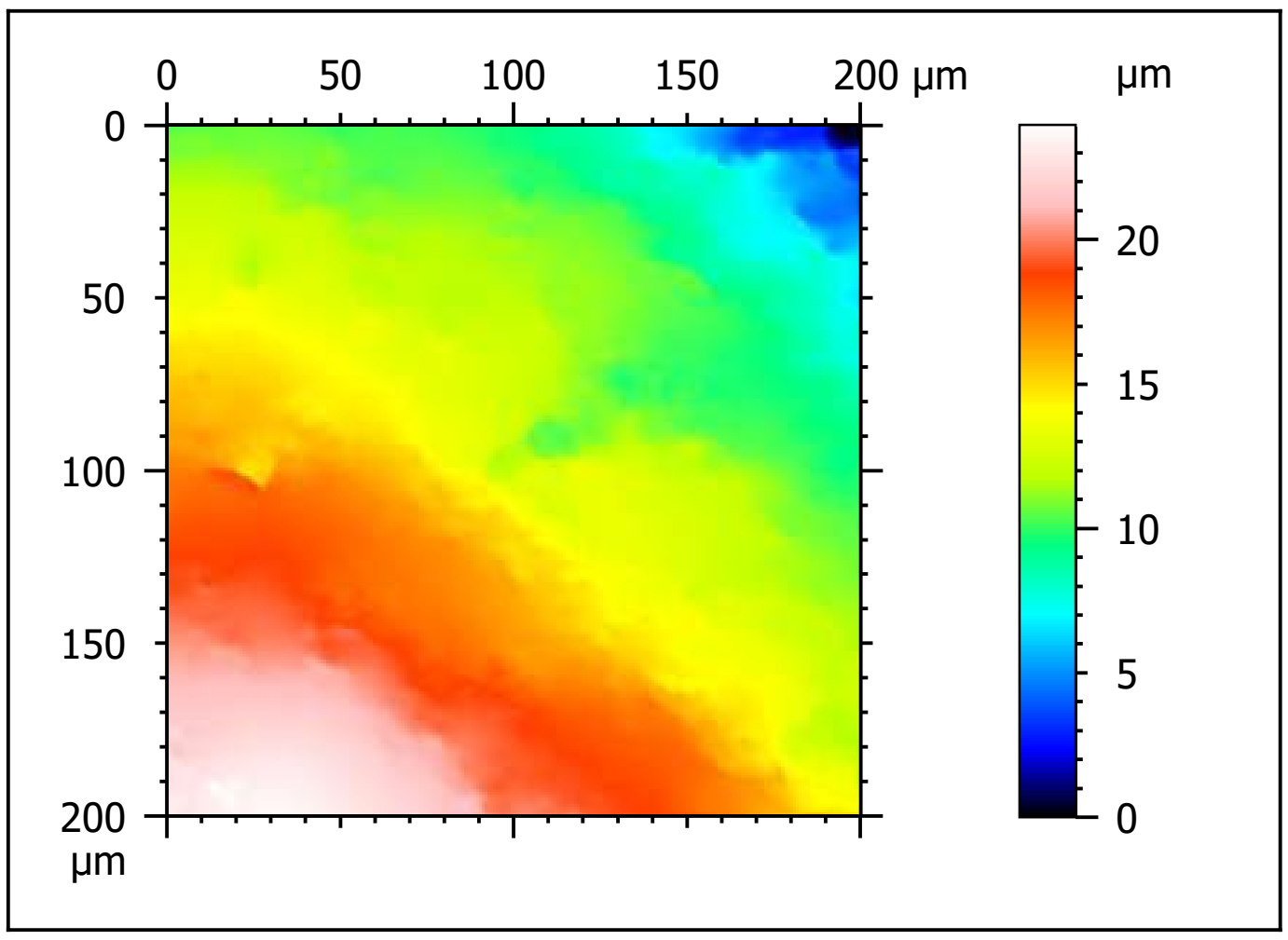

The Intensity and the topogrographic layers are separated and the later is kept

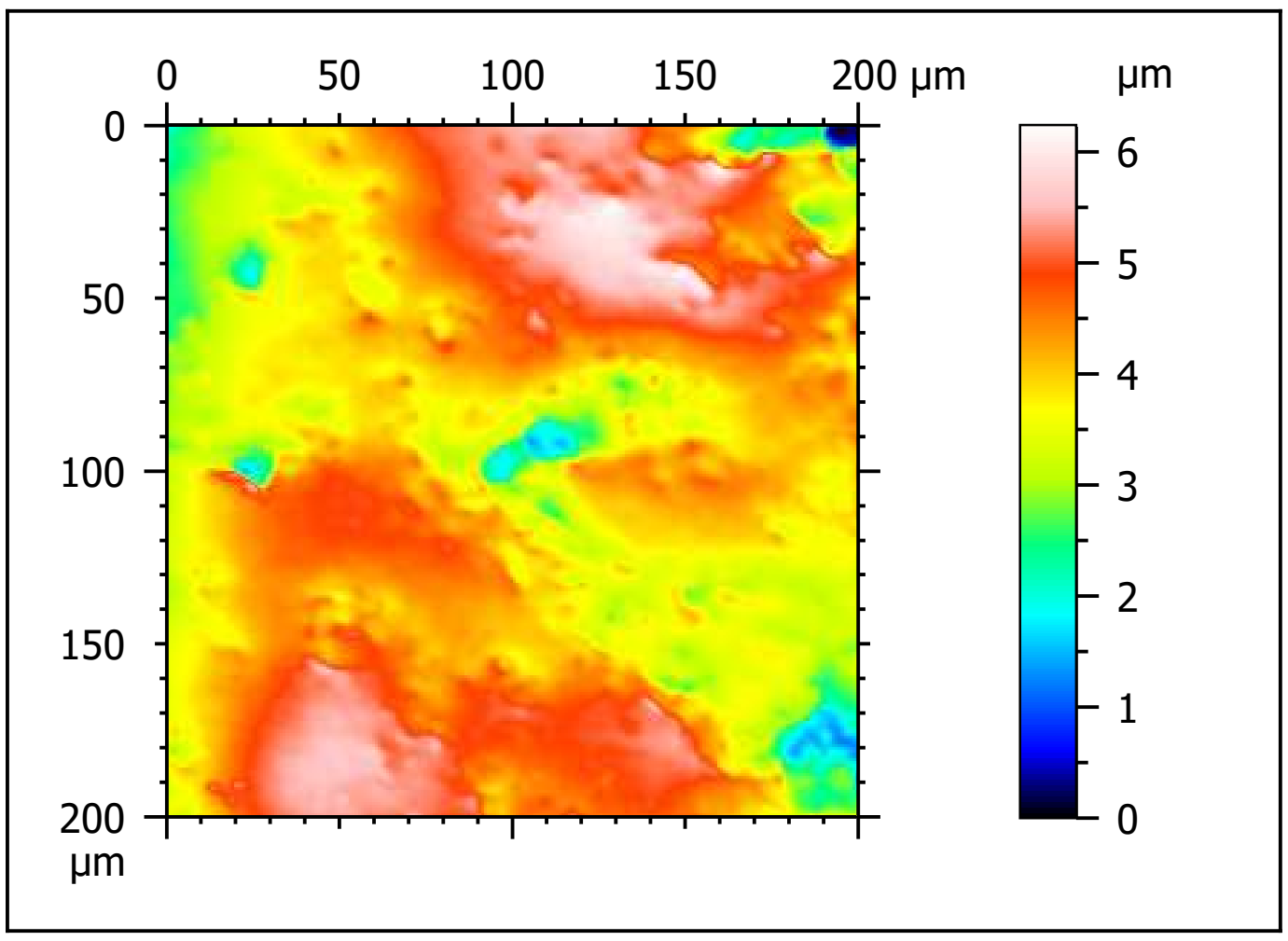

The topographic layer is levelled to get it flat, using the Least squares plane by rotation

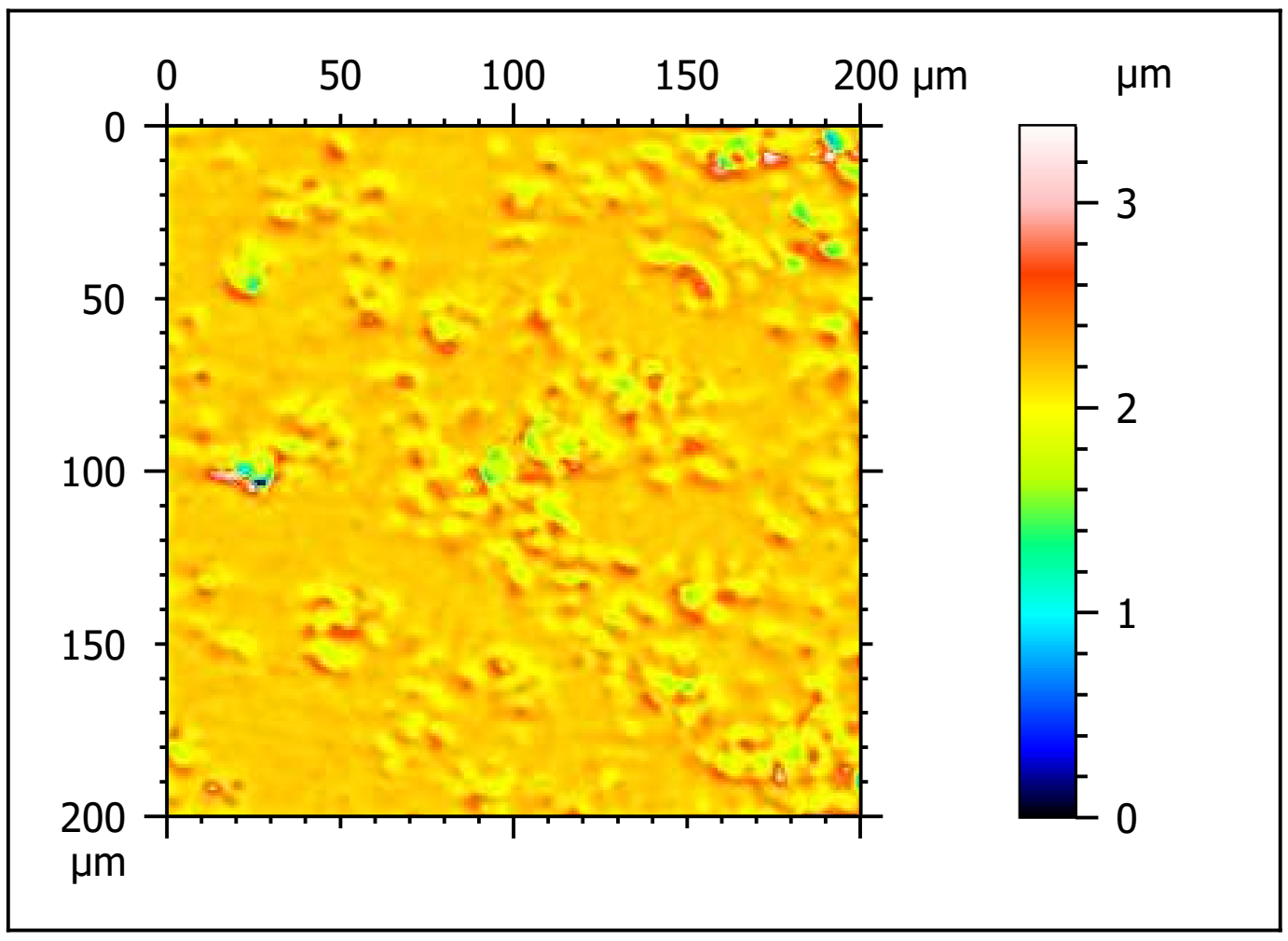

Filtering using the spatial filter with the Median (smooting) type, with a 13x13 filtering size

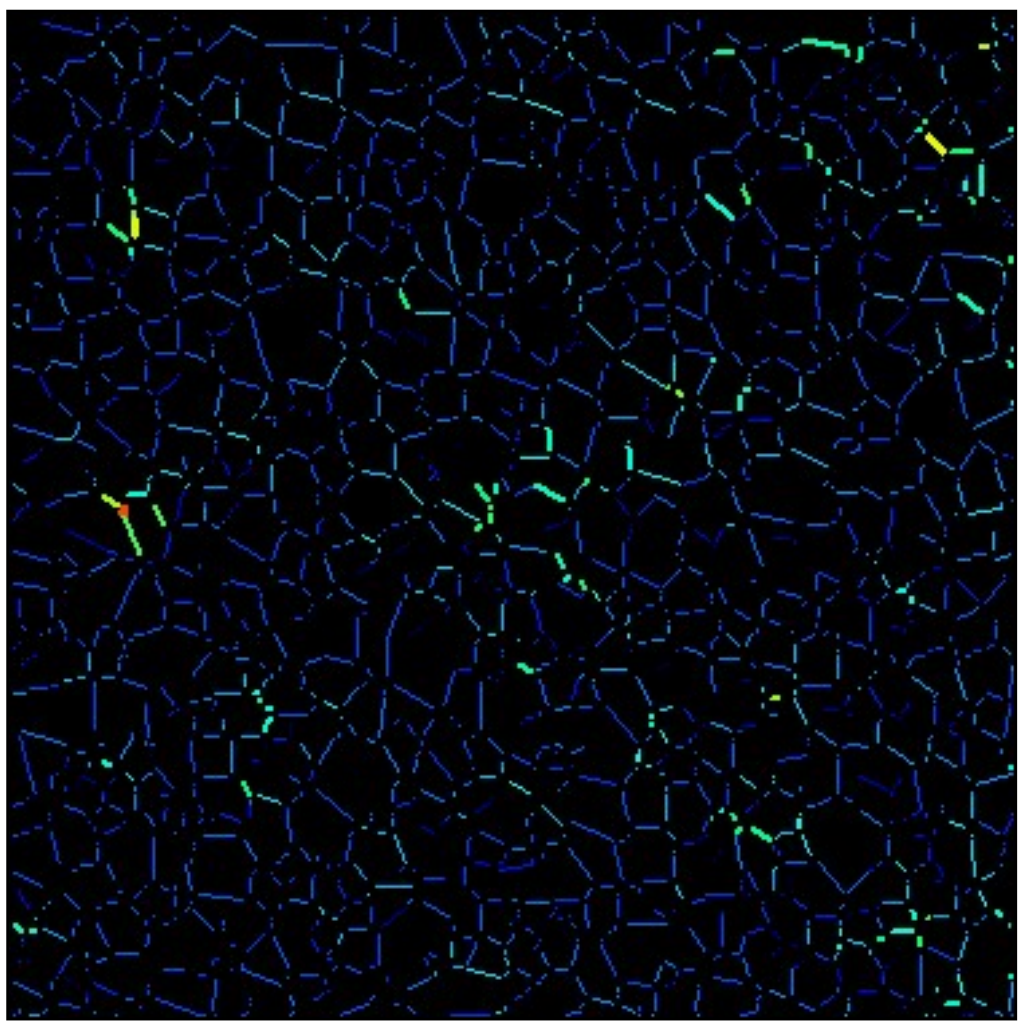

All furrows are shown.

Parameters	Value	Unit	
Maximum depth of furrows	1.01	µm	
Mean depth of furrows	0.219	µm	
Mean density of furrows	1195	cm/cm2	

Mesure of the surface using parameters concerning the micro-valleys network

ISO 25178		
**Height Parameters**		
Sq	0.140	µm
Ssk	-0.493	
Sku	17.3	
Sp	1.23	µm
Sv	2.15	µm
Sz	3.38	µm
Sa	0.0844	µm
**Functional Parameters**		
Smr	4.18	%
Smc	0.122	µm
Sxp	0.299	µm
**Spatial Parameters**		
Sal	2.26	µm
Str	0.458	
Std	149	°
**Hybrid Parameters**		
Sdq	0.108	
Sdr	0.544	%
**Functional Parameters (Vol...**		
Vm	0.0128	µm³/µm²
Vv	0.135	µm³/µm²
Vmp	0.0128	µm³/µm²
Vmc	0.0743	µm³/µm²
Vvc	0.112	µm³/µm²
Vvv	0.0229	µm³/µm²
**Feature Parameters**		
Spd	0.00332	1/µm²
Spc	0.211	1/µm
S10z	2.27	µm
S5p	0.974	µm
S5v	1.30	µm
Sda	290	µm²
Sha	255	µm²
Sdv	7.58	µm³
Shv	6.25	µm³
**Functional Parameters (Str...**		
Sk	0.148	µm
Spk	0.216	µm
Svk	0.193	µm
Smr1	16.3	%
Smr2	79.6	%
Spq	0.113	
Svq	0.251	
Smq	94.6	
**EUR 15178N**		
**Hybrid Parameters**		
Sds	0.0197	1/µm²
Ssc	0.0803	1/µm
Sfd	2.45	
**Area and Volume Parameters**		
Sdc	0.186	µm
**Functional Parameters**		
Sk	0.148	µm
Spk	0.216	µm
Svk	0.193	µm

EUR 15178N		
**Functional Parameters**		
Sr1	16.3	%
Sr2	79.6	%
Spq	0.113	
Svq	0.251	
Smq	94.6	
**Functional Indices**		
Sbi	0.137	
Sci	1.35	
Svi	0.163	
**ISO 12781**		
**Flatness Parameters**		
FLTt	0.109	µm
FLTp	0.0565	µm
FLTv	0.0526	µm
FLTq	0.0298	µm
**Other 3D Parameters**		
**Miscellaneous**		
Smean	-0.00589	µm
Sdar	40230	µm²
Spar	40012	µm²
**EUR 16145 EN**		
**Spatial Parameters**		
Stdi	0.652	

Mesure of the surface using
ISO texture parameters
