# Supplementary material for: Wild cereal harvesting ca. 14,000 years ago at Jeftelik (Western Syria)
Source: PLoS One. 2026 Nov 10;21(11):e0350364. doi: 10.1371/journal.pone.0350364 (PMC13750610; doi:10.1371/journal.pone.0350364)
Supplement: Supplementary file 3 [file pone.0350364.s006.docx]

Reviewer #2: The article reads well and is well organized. There is a major issue in the dataset (SI1 and SI2 do not have the same data for the archaeological artifacts), and I think the duration model made on cereals should not be applied to estimate the time of use for sickle blades interpreted as mixed or as reed/grass. I also think the manuscript should contain much more figures from the textural data rather than only images that we'd find in a classic traceological paper.

We acknowledge the careful and sound review of our paper carried out by the anonymous evaluator. We specially appreciate having checked the databases, detecting an important error in our selection of the database for the analysis of the intensity of use in Jeftelik.

I must emphasize that I am no specialist at all of Natufian period and archaeological context. My evaluation of the work will focus on the methodological and tribological part. From what I can read, the context is well developed, and I understood the necessary things about the Natufian period.

I found the paper well written, clear and well organized. I mainly have issues with the presentation of data and the data itself, as well as with the use of the models. I think that the current version of the discussion, which of course relies on the current version of the results, should be modified according to the new results that the authors might have when, hopefully, revising their results in light of my comments. I tried to write the comments in a clear and constructive way, please apologize if the tone used to be very clear might seem a bit direct.

We have slightly modified the Discussion and Conclusion sections in order to adapt them to the new calculations carried out on the correct database.

## Introduction

I think the introduction is OK about the archaeological period and context. I think, since the manuscript is mainly about use wear, that the introduction could be enlarged with a short review of the work that has been done with these methods. The end of the paragraph 3 is a bit short given the main focus of the submitted manuscript. I would integrate and modify accordingly the first three paragraphs of the current Methodology section into the introduction.

Now, we include the following paragraph about previous studies:

Previous use-wear analyses of harvesting tools from pre-Neolithic contexts, prior to the development of our quantitative approach based on confocal microscopy, relied primarily on the visual comparison of experimental and archaeological tools. Evidence from the lakeshore site of Ohalo II demonstrates that wild cereal harvesting was practiced as early as the Last Glacial Maximum, approximately 23,000 years ago (Groman-Yaroslavski et al., 2016).

The harvesting of wild cereals during the Natufian period represents a key stage in the long transition from plant gathering to cultivation in Southwest Asia. Use-wear studies of glossed flint blades indicate that Natufian groups regularly cut silica-rich plants, particularly wild cereals, using composite sickles. Unger-Hamilton's experimental work demonstrated that the characteristics of sickle gloss vary according to harvesting conditions and use intensity, and that glossed blades from Natufian and Early Neolithic contexts can be related to the harvesting of wild cereals and other grasses (Unger-Hamilton, 1991). Patricia Anderson's experimental cultivation and harvesting of wild einkorn further showed that cutting green or semi-ripe wild cereals produces distinctive microwear traces comparable to those observed on Natufian sickle blades. These experiments suggest that Natufian communities often harvested wild cereals before full ripening, when the grains were still attached to the rachis and could be collected efficiently before natural seed dispersal (Anderson, 1991).

## More recent quantitative texture analyses have confirmed that Natufian cereal harvesting mainly targeted wild cereals growing in natural stands (Ibáñez et al., 2021). These cereals were generally cut high on the stem, close to the ear rather than near the ground, indicating that the primary objective was grain collection rather than the systematic acquisition of straw (Ibáñez et al., 2025).

## Material

It is not very clear in this section if the analyzed samples are lunates or not. I can understand that the focus on lunates aims at confirming the Natufian attribution, but we don’t know if the analyzed samples are lunates. It is only with the beginning of the discussion that I completely understood that the samples are not lunates. I think it could be make clearer by stating in this Material section that lunates are armatures. Maybe the paragraph about lunates is not that important to be here in material since this is not the material studied in the manuscript.

We have included the phrase: Functional analysis of the lunates currently underway indicates that they were used as projectile inserts. ( Ibanez et al 2008).

What is the definition of a sickle blade, since 5 were identified as being made on blades, 6 on bladelets, and 1 on a flake. Is the definition only linked to the gloss on an edge? So the very definition of the tool is based on the visible usewear? From the figures 3-7, given the variety of shapes and size, it seems so : one glossy cutting edge makes a sickle blade. So of course all sickle blades will have high luster, because this is the characteristic to identify them in the bags of lithics. This seems to me like a circular reasoning.

Now, we include this explanation in the paper:

In traditional typological studies of Natufian and Neolithic lithic assemblages, flakes and blades displaying macroscopic gloss are generally classified as sickle blades. Use-wear analysis has refined this interpretation by demonstrating that macroscopic gloss is not exclusive to cereal harvesting but may also result from other technical activities, such as cutting other siliceous plants, use as threshing-sledge elements, or working mineral materials in the presence of water. Experimental studies have shown that macroscopic gloss develops on plant-cutting tools after relatively short periods of use (approximately one hour). Consequently, the presence of macroscopic gloss provides an efficient criterion for selecting tools used in plant cutting, although implements used only for very short periods are unlikely to develop visible gloss and therefore remain undetected. Subsequent microscopic use-wear analysis allows gloss produced by plant cutting to be distinguished from gloss resulting from other activities, such as plant scraping, use in threshing sledges, or the working of mineral materials in the presence of water.

About breakage: is the breakage intentional before use, or is it a consequence of use? Please clarify.

We examined the relationship between fractures and polish on the sickle blade samples. We observed that in 5 instances, fracturing occurred before use, while in 2 instances, it occurred after use. In the remaining 5 instances, the relationship between fracture and polish was unclear.

## Methodology

In its current form, the methodology cannot be replicated by other researchers, especially given the treatment in MountainsMap V.8. More details are given in previous articles, but we cannot be sure what is exactly the process used in this study (for example, is there a Gaussian filter applied, or is it the same filtering method as in reference 41 with the kernel matrix?). It would be very interesting to share a template from MountainsMap with explanations of each step before the calculation of roughness parameters. I can see you did it in Supplementary file 1 of reference 52 which is interesting. It could be interesting to include both a .mnt file for people having access to MountainsMap, and also a pdf with more details for other readers.

A .mnt file and a copy in pdf version (SI Fig and SI Fig Mnt file) with the template from MountainsMap with explanations of each step before the calculation of roughness parameters are now included as Supplementary Information.

I was surprised to see the use of spatial filtering in MountainsMap, which is supposed to be a denoising operator, to separate waviness from roughness as you explain in the text of the articles : “*To separate the polish texture from the irregularities of the flint surface, we applied* [*spatial filtering*](https://www.sciencedirect.com/topics/earth-and-planetary-sciences/spatial-filtering) *by moving a filtering matrix (called a kernel matrix) over the surface. The arithmetic mean operator consists of averaging each point with its 13x13 neighbouring points. The microtexture is calculated by subtracting the filtered surface from the source surface.”* (in ref 52 and similar text in ref 41 since I do not have the same details here in the submitted manuscript). This is not standard procedure to separate waviness and roughness. It is supposed to be done through the operator Metrological Filter, as you mention yourself in ref 40 : “*Processing was also used to separate polish texture from the irregularities of the flint surface in the sampled area, which can be considered as background noise. For this, different techniques can be used, such as the Fourier transform, which allows the waviness and roughness to be separated (Ibanez et al., 2013), though in this case we have resorted to spatial filtering, which is done by moving a small filtering matrix (called a kernel matrix) over the surface. The arithmetic mean operator consists in averaging each point with its 13 x 13 neighboring points. The micro-texture is calculated by subtracting the filtered surface from the source surface, and this microtexture is measured in our analysis.*” And as you did in Ibanez et al 2013.

The separation of the use-wear microtexture from the underlying flint topography can be achieved using different low-pass filtering approaches. In surface metrology, Gaussian filters implemented through the ISO 16610 metrological filter are the most widely used solution for separating waviness from roughness. However, linear spatial filters constitute an alternative implementation of the same general principle, in which local surface averaging is performed by convolution with a kernel matrix. In the present study, we employed an arithmetic mean spatial filter (13 × 13 kernel), whereby the low-frequency component of the surface is estimated by neighbourhood averaging and subsequently subtracted from the original surface to isolate the microtexture associated with use-wear. Although mathematically different from a Gaussian weighting function, both procedures aim to remove long-wavelength surface components while preserving the high-frequency texture relevant for functional analysis. The spatial filter was selected because it provides stable and reproducible results for polished flint surfaces and, importantly, ensures methodological consistency with our previous quantitative texture studies, in which the same procedure has been successfully applied to discriminate worked materials and harvesting regimes (Ibáñez et al., 2014, 2016, 2021; Pichon et al., 2021, 2023, 2025; Ibáñez et al., 2025).

Checking on this template in reference 52, I can see that you used a spatial filter for denoising with the median instead of the Arithmetic mean selected in the operator, while in the reference 52 (and other published papers) you mention Arithmetic mean. I think this should be made clearer or the template modified.

The use of the spatial filter with the median mean is explained in the Template SI Fig.

I would also strongly encourage the authors to share their raw measurements, especially given the creation of a set of reference for a decade now in this subject. It could certainly prove useful to other researcher, and would allow to replicate the results with other pretreatment in MountainsMap or other softwares.

Now, the dataset of the Jeftelik textural parameters includes all the parameters, in SI Table.

## Results

**Important**: I do not understand why the roughness values in Supplementary information 1 and 2 are not the same for the same archaeological artifacts. For example, there are 21 measurements for JTK08 DH906 UE36 in both SI1 and SI2, but the values are not the same:

|  | **Values from SI1 in ascendant order (for easy comparison) for each variable** | | |  | **Values from SI2 in ascendant order (for easy comparison) for each variable** | | |
| --- | --- | --- | --- | --- | --- | --- | --- |
| **Inventory number** | **Sa** | **Sz** | **Sal** |  | **Sa** | **Sz** | **Sal** |
| JTK08 DH906 UE36 | 0.104234902 | 2.781542921 | 2.283248294 |  | 0.131186118 | 2.862837919 | 2.230700537 |
| JTK08 DH906 UE36 | 0.106838223 | 2.895948918 | 2.308793069 |  | 0.131562827 | 2.899872918 | 2.280852459 |
| JTK08 DH906 UE36 | 0.107535109 | 3.440213903 | 2.310604229 |  | 0.132703726 | 3.146946911 | 2.553159807 |
| JTK08 DH906 UE36 | 0.108818914 | 3.589636898 | 2.314531597 |  | 0.134540378 | 3.444162903 | 2.560160195 |
| JTK08 DH906 UE36 | 0.108893338 | 3.780661893 | 2.317882825 |  | 0.136335704 | 3.468523902 | 2.580045887 |
| JTK08 DH906 UE36 | 0.11426821 | 3.830767892 | 2.323964971 |  | 0.139693258 | 3.508777901 | 2.583822443 |
| JTK08 DH906 UE36 | 0.116569133 | 4.052503885 | 2.351480895 |  | 0.142380293 | 3.584798899 | 2.585657819 |
| JTK08 DH906 UE36 | 0.119657687 | 4.068654885 | 2.577805089 |  | 0.146088578 | 3.685573896 | 2.589165492 |
| JTK08 DH906 UE36 | 0.120216471 | 4.307025878 | 2.589720394 |  | 0.14631693 | 3.751288894 | 2.605382833 |
| JTK08 DH906 UE36 | 0.120541331 | 4.378988876 | 2.596614177 |  | 0.146714613 | 3.914915889 | 2.632982157 |
| JTK08 DH906 UE36 | 0.124611527 | 4.693857867 | 2.645035306 |  | 0.148207952 | 4.461690874 | 2.647991912 |
| JTK08 DH906 UE36 | 0.128729212 | 4.814058864 | 2.656145809 |  | 0.153654792 | 4.864624862 | 2.655004221 |
| JTK08 DH906 UE36 | 0.13201134 | 5.372104848 | 2.656932641 |  | 0.159469576 | 4.967991859 | 2.667022275 |
| JTK08 DH906 UE36 | 0.132198478 | 5.594973842 | 2.669124517 |  | 0.16141203 | 5.018826858 | 2.674374689 |
| JTK08 DH906 UE36 | 0.132788695 | 5.690667839 | 2.674816948 |  | 0.165805548 | 5.336202849 | 2.687254552 |
| JTK08 DH906 UE36 | 0.134329724 | 5.889194833 | 2.676529941 |  | 0.168927116 | 5.356506849 | 2.703205639 |
| JTK08 DH906 UE36 | 0.135044807 | 6.118269827 | 2.711936054 |  | 0.170755984 | 5.415811847 | 2.724633736 |
| JTK08 DH906 UE36 | 0.140988889 | 7.406719791 | 2.726109021 |  | 0.170911744 | 6.951200803 | 2.732896278 |
| JTK08 DH906 UE36 | 0.142429656 | 8.455701761 | 2.734420439 |  | 0.171677155 | 7.79540378 | 2.747303322 |
| JTK08 DH906 UE36 | 0.144040816 | 9.737740725 | 2.782080431 |  | 0.171737662 | 8.863266749 | 2.791857174 |
| JTK08 DH906 UE36 | 0.175294391 | 10.79769269 | 2.787036676 |  | 0.220848028 | 10.02349672 | 2.814866302 |

The roughness values reported in Supplementary Information 1 and Supplementary Information 2 were not identical for the same archaeological artefacts because an incorrect dataset was inadvertently used to calculate the intensity-of-use model. The incorrect measurements resulted from a slightly different surface-processing procedure applied prior to the extraction of the texture parameters. The dataset has now been corrected. The same database of archaeological tools from Jeftelik is now used both for discriminating the type of harvested plant (wild cereals versus other plants) and for estimating the intensity of use. All texture parameters have been recalculated following the protocol described in the article and detailed in the Template, ensuring complete consistency between the two supplementary datasets.

I do not understand why the manuscript contains many figures similar to what is done in classical traceology (figures 3-7), while the manuscript mainly relies on textural measurements and there is not a single figure showing these values: the comparison of plant harvesting, the comparison between roughness parameters and types of sickle blades (5 on blades, 6 on bladelets, 1 on flake), the comparison of archaeological vs. ethnographic etc. I would also like to see some 3D images from roughness measurement and where the 200x200 µm are placed. And even for the figures 3-7, I think it would be useful for usewear specialists to have also images from the experimental samples to compare visually.

We think that the figures 3 to 7 are useful for the specialists in use-wear analysis. Following the suggestion of the reviewer, we include in the supplementary information (SI Fig) a folder with a 3D scan of one archaeological tool and the 200x200 µm samples.

Plotting myself the 4 parameters selected after QDA for the different groups, I can see that most of the Reeds from experiment 7 are exactly similar to Wild cereals, same for part of the Experiment 8, and that the Grass experiment is different only by Sal (and few outliers with Sz, which is the big problem of using Sz which is highly sensitive to outliers).

Currently the SI1 for Experimentations is not reproducible since we do not have access to the original dataset prior to the QDA-based selection of the 4 roughness parameters. It would be useful in the future if some other researchers want to reproduce or replicate your work.

This will be done for future research

### Harvesting intensity

In this part, I think there is **an important problem**: if the model is done with only cereal harvesting, it should be applied only to the archaeological samples previously identified as mainly used for cereal harvesting. Otherwise, it is like saying that the first part of the results are useless since it is the same to harvest cereals or reeds if you can apply the cereal model to tools used for harvesting reeds.

Now, the model for the intensity of use is only applied to the five tools clearly classified as wild cereal harvesting tools.

I think that the python script for Random Forest Regression should be shared so that other researchers can 1) reproduce these results and 2) replicate this type of research.

The phyton script for the intensity of use is shared in Mazzucco and Ibáñez, 2026

### Summary of my review of material, methods, and results:

In its current version, I think the manuscript has major issues:

1) The very definition of sickle blades seems to be circular: there is a high polish -> I select it for my subsample of sickle blades, be it on a bladelet, a blade, or a flake.
2) Lack of figures to show the textural results which is the core of the article. Plotting the experimental values, the four parameters do not seem to distinguish very well the groups.
3) Lack of comparison of the usewear based on the typology of the sickle blades (bladelet, blade, flake)
4) Problem with data different in SI1 and SI2. Maybe the result will be totally changed if they are checked?
5) Should not use the cereal model for archaeological samples attributed to reeds.

## Figures

The caption of figures must be largely extended and much more precise (figure 3-7 for example must be more detailed with the name of the samples, the explanation of the panels etc.)

The captions for Figures 3–7 have been extensively expanded and detailed with specific sample names and panel explanations, as requested.

Figure 1 OK for the location of the site. Add a North arrow.

A north arrow has been added to the map

Figure 2 : OK

Figure 3-7 : these are nice but the captions need details. It would also be very nice to have the same type of figure for experimental so that the use-wear specialist can compare experiment vs. archaeological.

We have added further details within the figure descriptions .

## Discussion

Based on all these issues, I read the discussion but I am not sure that the results will not see major changes when the problems I underlined are corrected. For example, in the first paragraph of the discussion : “*and their use was consistently more intensive than that of tools used to cut other plants.*”. But the authors did choose 12 artifacts in bags of lithics by choosing with their naked-eye the most polished samples. Can we really make this kind of interpretation on a subsample selected this way? And how is it possible to compare the duration of use with different plants when the experimental model is based on a single type of plants?

I find it also overstated to write that “these data indicate that cereal cutting was practiced, but not at a scale implying systematic wild cereal collection using a reaping technique” given that the authors subsamples the lithic material in a way to keep only the glossed tools. What if the tools were used much less time and therefore the gloss did not develop? Maybe, by showing some images of the experimental tools, the reader could see that even a few hours of cutting cereals produce a gloss? Here again the reasoning seems circular since the few objects analyzed tend to demonstrate a long use, but the selection of glossed artifacts may be a strong bias in favor of long use since the other artifacts have not been analyzed.

The discussion section has been adapted to the changes made in the calculations of the use intensity

## Bibliography and other minor comments

Ref 1: lack 2 out of 3 authors

I checked some other references randomly and did not find other errors.

I think all papers sent to reviewers should have line numbers to help reviewers do a better job.

Last paragraph of Experimental Reference Framework there is a problem in the middle with the remaining words of a previous sentence probably.

The text and the references have been modified according to the reviewer's comments.

We agree that line numbers are essential for an efficient review process. Accordingly, we have added line numbers throughout the "manuscript " to facilitate tracking the changes.
