# Supplementary material for: Wild cereal harvesting ca. 14,000 years ago at Jeftelik (Western Syria)
Source: PLoS One. 2026 Nov 10;21(11):e0350364. doi: 10.1371/journal.pone.0350364 (PMC13750610; doi:10.1371/journal.pone.0350364)
Supplement: Peer review history

# Peer review: Decision Letter 0

**DOI:** 10.1371/journal.pone.0350364.r001

**Authors:** Iris Groman-Yaroslavski

28 Jun 2026

Dear Dr. Abdo,

Thank you for submitting your manuscript to PLOS ONE. After careful consideration, we feel that it has merit but does not fully meet PLOS ONE’s publication criteria as it currently stands. Therefore, we invite you to submit a revised version of the manuscript that addresses the points raised during the review process.

Please submit your revised manuscript by Aug 27 2026 11:59PM. If you will need more time than this to complete your revisions, please reply to this message or contact the journal office at plosone@plos.org. When you're ready to submit your revision, log on to https://www.editorialmanager.com/pone/ and select the 'Submissions Needing Revision' folder to locate your manuscript file.

- A letter that responds to each point raised by the academic editor and reviewer(s). You should upload this letter as a separate file labeled 'Response to Reviewers'.
- A marked-up copy of your manuscript that highlights changes made to the original version. You should upload this as a separate file labeled 'Revised Manuscript with Track Changes'.
- An unmarked version of your revised paper without tracked changes. You should upload this as a separate file labeled 'Manuscript'.

If you would like to make changes to your financial disclosure, please include your updated statement in your cover letter. Guidelines for resubmitting your figure files are available below the reviewer comments at the end of this letter.

If applicable, we recommend that you deposit your laboratory protocols in protocols.io to enhance the reproducibility of your results. Protocols.io assigns your protocol its own identifier (DOI) so that it can be cited independently in the future. For instructions see: https://journals.plos.org/plosone/s/submission-guidelines#loc-laboratory-protocols. Additionally, PLOS ONE offers an option for publishing peer-reviewed Lab Protocol articles, which describe protocols hosted on protocols.io. Read more information on sharing protocols at https://plos.org/protocols?utm_medium=editorial-email&utm_source=authorletters&utm_campaign=protocols.

As the corresponding author, your ORCID iD is verified in the submission system and will appear in the published article. PLOS supports the use of ORCID, and we encourage all coauthors to register for an ORCID iD and use it as well. Please encourage your coauthors to verify their ORCID iD within the submission system before final acceptance, as unverified ORCID iDs will not appear in the published article. *Only*  the individual author can complete the verification step; PLOS staff *cannot*  verify ORCID iDs on behalf of authors.

We look forward to receiving your revised manuscript.

Kind regards,

Iris Groman-Yaroslavski, Ph.D

Academic Editor

PLOS One

**Journal Requirements:**

When submitting your revision, we need you to address these additional requirements.

1. Please ensure that your manuscript meets PLOS ONE's style requirements, including those for file naming. The PLOS ONE style templates can be found at

https://journals.plos.org/plosone/s/file?id=wjVg/PLOSOne_formatting_sample_main_body.pdf and

https://journals.plos.org/plosone/s/file?id=ba62/PLOSOne_formatting_sample_title_authors_affiliations.pdf

2. Please include a complete copy of PLOS’ questionnaire on inclusivity in global research in your revised manuscript. Our policy for research in this area aims to improve transparency in the reporting of research performed outside of researchers’ own country or community. The policy applies to researchers who have travelled to a different country to conduct research, research with Indigenous populations or their lands, and research on cultural artefacts. The questionnaire can also be requested at the journal’s discretion for any other submissions, even if these conditions are not met.  Please find more information on the policy and a link to download a blank copy of the questionnaire here: https://journals.plos.org/plosone/s/best-practices-in-research-reporting. Please upload a completed version of your questionnaire as Supporting Information when you resubmit your manuscript.

3. In your manuscript, please provide additional information regarding the specimens used in your study. Ensure that you have reported human remain specimen numbers and complete repository information, including museum name and geographic location.

If permits were required, please ensure that you have provided details for all permits that were obtained, including the full name of the issuing authority, and add the following statement:

'All necessary permits were obtained for the described study, which complied with all relevant regulations.'

If no permits were required, please include the following statement:

'No permits were required for the described study, which complied with all relevant regulations.'

For more information on PLOS One's requirements for paleontology and archeology research, see https://journals.plos.org/plosone/s/submission-guidelines#loc-paleontology-and-archaeology-research.

4. Please note that PLOS One has specific guidelines on code sharing for submissions in which author-generated code underpins the findings in the manuscript. In these cases, we expect all author-generated code to be made available without restrictions upon publication of the work. Please review our guidelines at https://journals.plos.org/plosone/s/materials-and-software-sharing#loc-sharing-code and ensure that your code is shared in a way that follows best practice and facilitates reproducibility and reuse.

5. Thank you for stating the following financial disclosure:

“Predoctoral contract funded by the Ministry of Science, Innovation and Universities, call: PID2022 of the AEI aid: PREP2022-000961; Project reference: PID2022-142075NB-I00”

Please state what role the funders took in the study.  If the funders had no role, please state: "The funders had no role in study design, data collection and analysis, decision to publish, or preparation of the manuscript."

If this statement is not correct you must amend it as needed.

Please include this amended Role of Funder statement in your cover letter; we will change the online submission form on your behalf.

6. We note that Figures 1 and 2 in your submission contain map images which may be copyrighted. All PLOS content is published under the Creative Commons Attribution License (CC BY 4.0), which means that the manuscript, images, and Supporting Information files will be freely available online, and any third party is permitted to access, download, copy, distribute, and use these materials in any way, even commercially, with proper attribution. For these reasons, we cannot publish previously copyrighted maps or satellite images created using proprietary data, such as Google software (Google Maps, Street View, and Earth). For more information, see our copyright guidelines: http://journals.plos.org/plosone/s/licenses-and-copyright.

We require you to either (1) present written permission from the copyright holder to publish these figures specifically under the CC BY 4.0 license, or (2) remove the figures from your submission:

1. You may seek permission from the original copyright holder of Figure(s) [#] to publish the content specifically under the CC BY 4.0 license.

We recommend that you contact the original copyright holder with the Content Permission Form (http://journals.plos.org/plosone/s/file?id=7c09/content-permission-form.pdf) and the following text:

“I request permission for the open-access journal PLOS ONE to publish XXX under the Creative Commons Attribution License (CCAL) CC BY 4.0 (http://creativecommons.org/licenses/by/4.0/). Please be aware that this license allows unrestricted use and distribution, even commercially, by third parties. Please reply and provide explicit written permission to publish XXX under a CC BY license and complete the attached form.”

Please upload the completed Content Permission Form or other proof of granted permissions as an "Other" file with your submission.

In the figure caption of the copyrighted figure, please include the following text: “Reprinted from [ref] under a CC BY license, with permission from [name of publisher], original copyright [original copyright year].”

2. If you are unable to obtain permission from the original copyright holder to publish these figures under the CC BY 4.0 license or if the copyright holder’s requirements are incompatible with the CC BY 4.0 license, please either i) remove the figure or ii) supply a replacement figure that complies with the CC BY 4.0 license. Please check copyright information on all replacement figures and update the figure caption with source information. If applicable, please specify in the figure caption text when a figure is similar but not identical to the original image and is therefore for illustrative purposes only.

The following resources for replacing copyrighted map figures may be helpful:

USGS National Map Viewer (public domain): http://viewer.nationalmap.gov/viewer/

The Gateway to Astronaut Photography of Earth (public domain): http://eol.jsc.nasa.gov/sseop/clickmap/

Maps at the CIA (public domain): https://www.cia.gov/library/publications/the-world-factbook/index.html and https://www.cia.gov/library/publications/cia-maps-publications/index.html

NASA Earth Observatory (public domain): http://earthobservatory.nasa.gov/

Landsat: http://landsat.visibleearth.nasa.gov/

USGS EROS (Earth Resources Observatory and Science (EROS) Center) (public domain): http://eros.usgs.gov/#

Natural Earth (public domain): http://www.naturalearthdata.com/

7. We note that Figures 2 – 8 in your submission contain copyrighted images. All PLOS content is published under the Creative Commons Attribution License (CC BY 4.0), which means that the manuscript, images, and Supporting Information files will be freely available online, and any third party is permitted to access, download, copy, distribute, and use these materials in any way, even commercially, with proper attribution. For more information, see our copyright guidelines: http://journals.plos.org/plosone/s/licenses-and-copyright.

We require you to either (1) present written permission from the copyright holder to publish these figures specifically under the CC BY 4.0 license, or (2) remove the figures from your submission:

1. You may seek permission from the original copyright holder of Figures 2 - 8 to publish the content specifically under the CC BY 4.0 license.

We recommend that you contact the original copyright holder with the Content Permission Form (http://journals.plos.org/plosone/s/file?id=7c09/content-permission-form.pdf) and the following text:

“I request permission for the open-access journal PLOS ONE to publish XXX under the Creative Commons Attribution License (CCAL) CC BY 4.0 (http://creativecommons.org/licenses/by/4.0/). Please be aware that this license allows unrestricted use and distribution, even commercially, by third parties. Please reply and provide explicit written permission to publish XXX under a CC BY license and complete the attached form.”

Please upload the completed Content Permission Form or other proof of granted permissions as an "Other" file with your submission.

In the figure caption of the copyrighted figure, please include the following text: “Reprinted from [ref] under a CC BY license, with permission from [name of publisher], original copyright [original copyright year].”

2. If you are unable to obtain permission from the original copyright holder to publish these figures under the CC BY 4.0 license or if the copyright holder’s requirements are incompatible with the CC BY 4.0 license, please either i) remove the figure or ii) supply a replacement figure that complies with the CC BY 4.0 license. Please check copyright information on all replacement figures and update the figure caption with source information. If applicable, please specify in the figure caption text when a figure is similar but not identical to the original image and is therefore for illustrative purposes only.

8. Please include captions for your Supporting Information files at the end of your manuscript, and update any in-text citations to match accordingly. Please see our Supporting Information guidelines for more information: http://journals.plos.org/plosone/s/supporting-information.

9. If the reviewer comments include a recommendation to cite specific previously published works, please review and evaluate these publications to determine whether they are relevant and should be cited. There is no requirement to cite these works unless the editor has indicated otherwise.

**Additional Editor Comments:**

The article is of high importance as it deals with the issue of cereal harvesting in a period much earlier than cereal domestication. However, some structural changes are needed, in data and structure of the MS but especially a more proportional presentation of the data and results. This was observed by the reviewers and need to be addressed with care. In addition, a more thorough review of what we know of this subject is needed with the latest references.

[Note: HTML markup is below. Please do not edit.]

Reviewers' comments:

Reviewer's Responses to Questions

**Comments to the Author**

1. Is the manuscript technically sound, and do the data support the conclusions?

Reviewer #1: Yes

Reviewer #2: Partly

**********

2. Has the statistical analysis been performed appropriately and rigorously?

Reviewer #1: Yes

Reviewer #2: Yes

**********

3. Have the authors made all data underlying the findings in their manuscript fully available?

The PLOS Data policy

Reviewer #1: Yes

Reviewer #2: No

**********

4. Is the manuscript presented in an intelligible fashion and written in standard English?

Reviewer #1: Yes

Reviewer #2: Yes

**********

Reviewer #1: This is an important study that significantly advances our understanding of harvesting practices prior to cereal domestication. It highlights the value of examining small assemblages from sites where cereals may not have played a major role in subsistence. I hope that my comments will help to further improve this manuscript.

The abstract is adequate and summarises the manuscript well. The introduction is well structured and provides a clear overview of the Natufian role in plant exploitation.

Site

Is it necessary to provide the full description of the reduction strategies and all associated details? How does the detailed description of the tools contribute to, or integrate with, the main arguments of the article? The article focuses on quantifying a small number of sickle blades from the site and doesn’t discuss other technological issues related to the tools’ production. In my opinion, a short paragraph summarising the lithic technology as background to the sickle-blades description would be sufficient.

In the introduction, the authors state that sickle-blades constitute 1% of the lithic assemblage, whereas in this section, we are told they represent 1.07% of the retouched tool assemblage. This implies that sickle-blades, in fact, make up less than 1% of the overall lithic assemblage. Furthermore, the raw counts underlying these percentages are not reported in the article.

Methods

“While traditional use-wear analysis relies on direct comparative examination, these newer quantitative approaches allow for a more precise characterization of worked materials.” The outcome of quantitative classifications is highly contingent upon the quality of the experimental datasets, cleaning methods and raw material type. This sentence and the following paragraph represent a broad generalisation. The first 3 paragraphs of the methodology section are superfluous, as they conflate research history with very general assertions about qualitative and quantitative approaches to use-wear. I fail to see their relevance to the current topic under discussion in this manuscript.

“In this study we have applied a technical procedure previously used in other studies to identify cereal cutting in different states of maturity (domestic-ripe; wild cultivated-semi ripe; wild in natural stands-green) and reeds [40], to which we added grass in a following paper [41].” Does it mean that the authors rely on previous experimental datasets and conduct additional experiments focusing on harvesting reeds, or use the same tools to mix traces of grass harvesting? Table 2 reports a small experimental dataset, with one implement used for grass cutting, so I am assuming that’s the case, but the sentence could be rewritten for clarity.

“It should be noted that the identification of the duration of use is approximate, as two main aspects differentiate the experimental reference collection from the archaeological one. First, the experimental program was carried out on fully ripe domesticated cereals, whereas the archaeological tools were likely used for harvesting wild cereals in natural stands before the plants reached full maturity. “ This is an important issue that should be discussed in greater depth, as it will influence all interpretations presented in the article. Were any experiments conducted to compare trace formation times between wild and domesticated cereals? If not, are there any publications reporting on that issue? The authors report that the Natufian sickle blades were interpreted as having been used to harvest wild cereals, presumably before ripening. The dryness of the stems would affect the rate of trace formation, as drier stems are more abrasive. Additionally, wild cereals could be growing in less-dense fields, which would also affect the harvesting time. The roughness measurements are surely helpful in interpreting the intensity of polish development, but making direct assumptions about the harvesting length without an experiment dedicated to wild cereal harvesting seems overstretched.

Experimental Reference Framework

“Consequently, harvesting experiments involving domesticated cereals or cultivated wild cereals were not included in the reference collection.” Except for the one on the duration of use reported above and in the last paragraph of this section.

“The cereal was in the hard-dough stage of ripening. the tool.” Please check the text for grammar and consistency.

Technical procedure

“From each acquisition, between one and four subsamples of 200 × 200 μm were extracted, which corresponds to the standard size used in our previous methodology to discriminate plant-related cutting polishes” How many measurements per tool in total?

Results

“The remaining six tools (1, 2, 3, 9, 10, and 11), display more balanced proportions, suggesting mixed plant processing activities (Fig 6 and 7). Although wild cereal cutting predominates in Tools 1, 3, and 11 (approximately 60–62%), a substantial proportion of samples is assigned to other plants, indicating that cereals were important but not exclusive targets. Tools 9 and 10 show near-equivalent distributions between the two categories, reinforcing the interpretation of polyvalent use (Table 4).” This is a very interesting finding that would be much stronger if the authors conducted experiments aimed to replicate more flexible sickle use, for example, using the same tools to harvest cereals and reeds. At this stage, the results are showing inconsistency in classifying use traces on the archaeological materials, but it could be argued that it comes from the discrepancies between the experimental and archaeological materials used to train the models, not necessarily from mixed plant use.

“The predictive model of harvesting intensity was developed using a controlled experimental reference consisting of 36 hours of domestic wheat harvesting, during which silicone imprints of the use-wear traces were taken at two-hour intervals [43]. Harvesting intensity was operationalized as duration of use (in hours).” This sentence belongs to the methods part.

“The model shows high predictive performance (R² ≈ 0.80– 0.83), indicating a strong relationship between surface texture parameters and use duration, with prediction errors typically within ±3–5 hours.” An error of 5 hours would significantly affect the times reported in Table 4. Maybe this should be reported in the table or included more in the interpretation.

“For each tool, all surface predictions were averaged, and the mean value was then assigned to one of the four intensity groups” is statistically acceptable, but it should be clear that this is a post hoc interpretive classification, not a prediction model output itself. If possible, the authors could report uncertainty or the distribution of surface-level predictions rather than only the mean.

“Crossing the classification of the type of plant harvested and the intensity of use shows a mean intensity of use of 18.6 hours for wild cereal cutting tools, 13.6 hours for the tools cutting wild cereals and other plants, and 9.2 hours for the tool used in cutting plants other than wild cereals. Then, tools used for cutting wild cereals were used more intensively than those used for cutting other plants.” I feel that, considering both the experimental constraints and the classification error, the same argument could be made without providing so precise hour measurements and instead keeping the use-duration distribution proposed earlier by the authors: low, medium, high, and very high.

Discussion

“Together, these data indicate that cereal cutting was practiced, but not at a scale implying systematic wild cereal collection using a reaping technique.” and “Taken together, the evidence indicates that wild cereals were indeed exploited during the Natufian period, but that sickle harvesting constituted only a minor component of a broader and more flexible plant-procurement system. “ Are any plant remains recorded at the site, charcoal or seed assemblages? If so, mentioning cereal occurrences vs other plant remains would strengthen this argument.

Table 5 – the dating for El-Wad Terrace can be found in publications. For example: Reuven Yeshurun, Elisabetta Boaretto, Ma’ayan Lev, Meir Orbach, Catherine Ujma, Sigal Lavy-Elbaz, Linda Amos, Mina Weinstein-Evron, A Natufian demographic cycle at el-Wad Terrace, Israel: The rise and fall of the architectural compound, Archaeological Research in Asia, Volume 41, 2025, 100599, ISSN 2352-2267, https://doi.org/10.1016/j.ara.2025.100599.

Conclusions

“This variability suggests that plant-cutting activities were not uniform but structured around different use contexts and durations.” Different use contexts were not discussed in the text, and, in my opinion, the small assemblage doesn’t allow for such a conclusion.

Reviewer #2: The article reads well and is well organized. There is a major issue in the dataset (SI1 and SI2 do not have the same data for the archaeological artifacts), and I think the duration model made on cereals should not be applied to estimate the time of use for sickle blades interpreted as mixed or as reed/grass. I also think the manuscript should contain much more figures from the textural data rather than only images that we'd find in a classic traceological paper.

Please find my review in the attached document.

**********

what does this mean?). If published, this will include your full peer review and any attached files.

If you choose “no”, your identity will remain anonymous but your review may still be made public.

**Do you want your identity to be public for this peer review?**  For information about this choice, including consent withdrawal, please see our Privacy Policy

Reviewer #1: No

Reviewer #2: **Yes:**  Alain Queffelec

**********

[NOTE: If reviewer comments were submitted as an attachment file, they will be attached to this email and accessible via the submission site. Please log into your account, locate the manuscript record, and check for the action link "View Attachments". If this link does not appear, there are no attachment files.]

To ensure your figures meet our technical requirements, please review our figure guidelines: https://journals.plos.org/plosone/s/figures

You may also use PLOS’s free figure tool, NAAS, to help you prepare publication quality figures: https://journals.plos.org/plosone/s/figures#loc-tools-for-figure-preparation.

NAAS will assess whether your figures meet our technical requirements by comparing each figure against our figure specifications.

Attachment Submitted filename: Review_AQ.docx

# Author comment: Author response to Decision Letter 1

**DOI:** 10.1371/journal.pone.0350364.r002

11 Sep 2026

Dear Editor and Reviewers,

We sincerely acknowledge the relevant, constructive, and insightful comments made by the Reviewers. Their feedback has been incredibly helpful and accurate, significantly contributing to improving the overall quality and clarity of our manuscript. We truly appreciate the time and effort dedicated to this careful and sound review.We have addressed all the points raised in the revision process. For convenience, the detailed point-by-point responses and clarifications have been uploaded as two separate files, corresponding to the comments of each individual reviewer.

Thank you once again for your valuable guidance and support throughout this process.

Attachment Submitted filename: Response to Reviewers 2.docx

# Peer review: Decision Letter 1

**DOI:** 10.1371/journal.pone.0350364.r003

**Authors:** Iris Groman-Yaroslavski

7 Oct 2026

Wild Cereal Harvesting ca. 14,000 Years Ago at Jeftelik (Western Syria).

PONE-D-26-22575R1

Dear Dr. Khaled Abdo,

We’re pleased to inform you that your manuscript has been judged scientifically suitable for publication and will be formally accepted for publication once it meets all outstanding technical requirements.

Within one week, you’ll receive an e-mail detailing the required amendments. When these have been addressed, you’ll receive a formal acceptance letter and your manuscript will be scheduled for publication.

An invoice will be generated when your article is formally accepted. Please note, if your institution has a publishing partnership with PLOS and your article meets the relevant criteria, all or part of your publication costs will be covered. Please make sure your user information is up-to-date by logging into Editorial Manager at Editorial Manager® and clicking the ‘Update My Information' link at the top of the page. For questions related to billing, please contact billing support.

If your institution or institutions have a press office, please notify them about your upcoming paper to help maximize its impact. If they’ll be preparing press materials, please inform our press team as soon as possible -- no later than 48 hours after receiving the formal acceptance. Your manuscript will remain under strict press embargo until 2 pm Eastern Time on the date of publication. For more information, please contact onepress@plos.org.

Kind regards,

Iris Groman-Yaroslavski, Ph.D

Academic Editor

PLOS One

Additional Editor Comments (optional):

Reviewers' comments:

Reviewer's Responses to Questions

**Comments to the Author**

Reviewer #1: All comments have been addressed

Reviewer #2: All comments have been addressed

**********

2. Is the manuscript technically sound, and do the data support the conclusions?

Reviewer #1: Yes

Reviewer #2: Yes

**********

3. Has the statistical analysis been performed appropriately and rigorously?

Reviewer #1: Yes

Reviewer #2: Yes

**********

4. Have the authors made all data underlying the findings in their manuscript fully available?

The PLOS Data policy

Reviewer #1: Yes

Reviewer #2: No

**********

5. Is the manuscript presented in an intelligible fashion and written in standard English?

Reviewer #1: Yes

Reviewer #2: Yes

**********

Reviewer #1: This is a very interesting article that broadens our understanding of cereal use by the Natufian groups, and the authors have made significant improvements to the manuscript. The notion that sickle blades were used to harvest cereals alongside other plants, such as reeds and grasses, is important and well suited to activities involving undomesticated plants. Overall, the article presents an interesting take on the potential cereal use, and the discussion provides important examples of alternative cereal harvesting. I have only minor comments and believe the article can be published in its current form.

In the future, when applying a similar approach to interpreting plant traces, the authors could consider several issues. The interpretations presented in the text could be significantly strengthened if the authors conducted more experiments replicating the conditions in which cereal use is mixed with other traces within one tool. At this stage, the potential influence of each plant on the joint trace remains unassessed, and the interpretation instead relies on mixed fitting of points measured within one tool. Additionally, the length of tool use would significantly influence trace development and should therefore be factored into the assessment using the experimental comparatives. In my opinion, the discussion should include factors that may affect the reliability of the classification.

Some final editing will be needed before the article is published. There appears to be an issue with the reference formatting. Numerous references in the text now show as “Error! Reference source not found”.

Lines 284 to 287: “Consequently, harvesting experiments involving domesticated cereals or cultivated wild cereals were not included in the reference collection. The cereal cutting experiments used in this study therefore consist exclusively of the harvesting of wild cereals in natural stands” – I still find this sentence confusing, as part of this study is clearly based on the harvesting time estimates resulting from experimental harvesting of domesticated cereals. I understand that the authors are differentiating between the experimental tools presented in Table 2 and those discussed earlier in the text. Nevertheless, this split makes the procedure less clear.

Reviewer #2: Dear authors,

thank you for considering my comments on the first version of your manuscript.

I must emphasize that when I was asking for data sharing, I was not talking about the table of all the results for the measurements on archaeological materials (which is already very important) but for the complete dataset of .mnt files of the reference collection. This would ensure that other researchers can use your reference data for their own research (with other pre-treatments, other analysis in Mountains etc.). This could be made here or, in a specific datapaper which would be very interesting for the community.

I still think figures including plots of quantitative data would be welcomed in the paper, instead of only tables for quantitative data, and images fro qualitative traceology.

Very minor modification: I think there are 265 measured surface in the experimental used to model the intensity of use, when you wrote 264 in the paper just before Table 3.

**********

what does this mean?). If published, this will include your full peer review and any attached files.

If you choose “no”, your identity will remain anonymous but your review may still be made public.

**Do you want your identity to be public for this peer review?**  For information about this choice, including consent withdrawal, please see our Privacy Policy

Reviewer #1: No

Reviewer #2: **Yes:**  Alain Queffelec

**********

# Editor report: Acceptance letter

**DOI:** 10.1371/journal.pone.0350364.r004

**Authors:** Iris Groman-Yaroslavski

PONE-D-26-22575R1

PLOS One

Dear Dr. Abdo,

I'm pleased to inform you that your manuscript has been deemed suitable for publication in PLOS One. Congratulations! Your manuscript is now being handed over to our production team.

At this stage, our production department will prepare your paper for publication. This includes ensuring the following:

* All references, tables, and figures are properly cited

* All relevant supporting information is included in the manuscript submission,

* There are no issues that prevent the paper from being properly typeset

You will receive further instructions from the production team, including instructions on how to review your proof when it is ready. Please keep in mind that we are working through a large volume of accepted articles, so please give us a few days to review your paper and let you know the next and final steps.

Lastly, if your institution or institutions have a press office, please let them know about your upcoming paper now to help maximize its impact. If they'll be preparing press materials, please inform our press team within the next 48 hours. Your manuscript will remain under strict press embargo until 2 pm Eastern Time on the date of publication. For more information, please contact onepress@plos.org.

You will receive an invoice from PLOS for your publication fee after your manuscript has reached the completed accept phase. If you receive an email requesting payment before acceptance or for any other service, this may be a phishing scheme. Learn how to identify phishing emails and protect your accounts at https://explore.plos.org/phishing.

If we can help with anything else, please email us at customercare@plos.org.

Thank you for submitting your work to PLOS One and supporting open access.

Kind regards,

PLOS One Editorial Office Staff

on behalf of

Dr. Iris Groman-Yaroslavski

Academic Editor

PLOS One
